# Habitat availability explains variation in climate-driven range shifts across multiple taxonomic groups

**DOI:** 10.1038/s41598-019-51582-2

**Published:** 2019-10-21

**Authors:** Philip J. Platts, Suzanna C. Mason, Georgina Palmer, Jane K. Hill, Tom H. Oliver, Gary D. Powney, Richard Fox, Chris D. Thomas

**Affiliations:** 1 Department of Environment and Geography, University of York, Wentworth Way, York, YO10 5NG UK; 2 Department of Biology, University of York, Wentworth Way, York, YO10 5DD UK; 3 NERC Centre for Ecology and Hydrology, Wallingford Oxfordshire, OX10 8BB UK; 4 School of Biological Sciences, University of Reading, Reading, Berkshire, RG6 6AS UK; 5 Butterfly Conservation, Manor Yard, East Lulworth, Wareham, Dorset, BH20 5QP UK

**Keywords:** Biodiversity, Climate-change ecology, Population dynamics, Entomology

## Abstract

Range shifting is vital for species persistence, but there is little consensus on why individual species vary so greatly in the rates at which their ranges have shifted in response to recent climate warming. Here, using 40 years of distribution data for 291 species from 13 invertebrate taxa in Britain, we show that interactions between habitat availability and exposure to climate change at the range margins explain up to half of the variation in rates of range shift. Habitat generalists expanded faster than more specialised species, but this intrinsic trait explains less of the variation in range shifts than habitat availability, which additionally depends on extrinsic factors that may be rare or widespread at the range margin. Similarly, while climate change likely underlies polewards expansions, we find that more of the between-species variation is explained by differences in habitat availability than by changes in climatic suitability. A model that includes both habitat and climate, and their statistical interaction, explains the most variation in range shifts. We conclude that climate-change vulnerability assessments should focus as much on future habitat availability as on climate sensitivity and exposure, with the expectation that habitat restoration and protection will substantially improve species’ abilities to respond to uncertain future climates.

## Introduction

Many species are shifting their distributions polewards and to higher elevations in response to climate warming^1–3^, but there is extremely large variation in the rates at which the range boundaries of individual species are moving^4–8^. This variation could arise from differences in climate sensitivity, resource requirements, reproductive rates, phenotypic plasticity, dispersal ability or biotic interactions^9–15^. Meta-analyses show that species’ traits related to habitat specialisation are the most consistent predictors of variation in range shift^5,7^. However, no combination of intrinsic traits can explain a large proportion of the variation across multiple taxonomic groups – likely due to trait interactions with extrinsic factors in range-margin landscapes^5^. For example, the role of habitat specialisation in facilitating or inhibiting range shifts is contingent on whether a species is specialised on habitats that are common or rare at the range margin, and on the abundance of required resources within those habitat classes. Given that landscape conditions vary, both geographically and from the perspectives of species with different resource requirements, the extent to which habitat associations underlie the observed variation in recent range shifts, relative to species- and group-level differences in climate sensitivity and exposure, remains unknown.

The ‘habitat’ of any species depends on many interacting factors, including physical aspects of the environment (such as geology), the nature of the vegetation (e.g., influencing microclimates), directly used resources (including host-plant densities for herbivorous insects), and sufficient capacity to escape from predators, diseases and other natural enemies. Thus, the capacity of a species to utilise any particular land cover (a.k.a. ecosystem or vegetation type) could be limited by multiple factors, and different limitations may occur in different vegetation types, and in different locations within a given type. Nonetheless, it remains useful to compare the habitat breadth (specialisation to generalisation) of a set of species across a range of recognised vegetation types, and thus compare habitat availability at species’ range margins with observed rates of range shift. This approach allows a wide range of species to be considered, particularly in Britain which has some of the largest datasets of species occurrence records in the world.

Here, using 25 million hectare-resolution occurrence records for invertebrate species in mainland Britain, we show that habitat-climate interactions in species’ range margins explain up to half of the observed variation in rates of range shift across 291 species in 13 taxonomic groups (aquatic bugs, bees, butterflies, dragonflies and damselflies, grasshoppers and allies, ground beetles, hoverflies, macromoths, non-marine molluscs, shieldbugs and allies, soldierflies and allies, spiders, and wasps; Table S1).

## Results

### Range shifts

**Figure 1:**
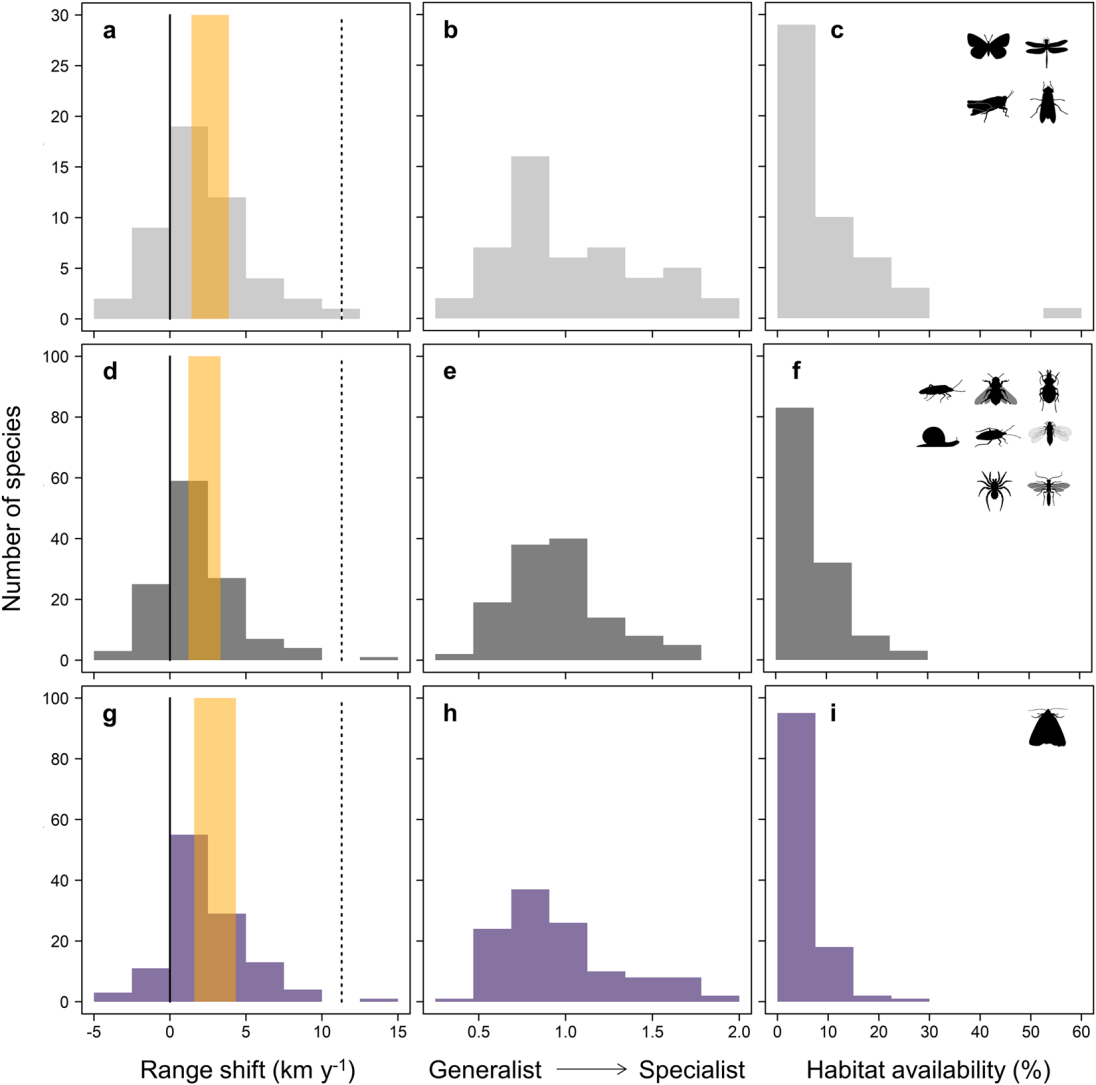
Latitudinal range-margin shift, habitat specialisation, and habitat availability in species’ range margins. (**a**–**c**), Four taxonomic groups with high recording intensity (butterflies, dragonflies and damselflies, grasshoppers and allies, and hoverflies; n = 49 species). (**d**–**f**), Eight groups with lower recording intensity (n = 126 species). (**g**–**i**), Macromoths (different recording method, n = 116 species). In a, d and g: solid lines show zero shift, orange bars show 95% CIs for mean observed shifts, and dotted lines show the shift in mean annual isotherms given observed warming between the two recording periods (1976–1990 and 2001–2015; 11.2 km y^−1^, Fig. S1).

Each species considered here reaches its northern (poleward) range margin in Britain and might, therefore, be expected to expand northward during a period of sustained regional warming. We measured range shifts (latitudinal changes in the ten-northernmost occupied 10 km × 10 km grid squares) between 1976–1990 and 2001–2015, during which time the mean annual temperature of the study region warmed by 0.8 °C (Fig. S1). The mean observed range shift was 51 km northwards at a rate of 2 km y^−1^ (95% CI: [1.4, 3.8]). This is similar to rates of range shift reported previously for British fauna^16,17^, and is similar to or faster than rates reported globally^1,16^. We found considerable variation among species (Fig. 1 and Table S2), with nearly all of this variation occurring within, rather than among, taxonomic groups (R^2^ = 2% in a random intercept model of range shift *vs*. group). Thus, major trait differences among groups cannot be responsible for the large variation in range shifts. In contrast, individualistic attributes of species and/or location-specific constraints, such as habitat, could still make strong contributions.

### Habitat associations

**Figure 2:**
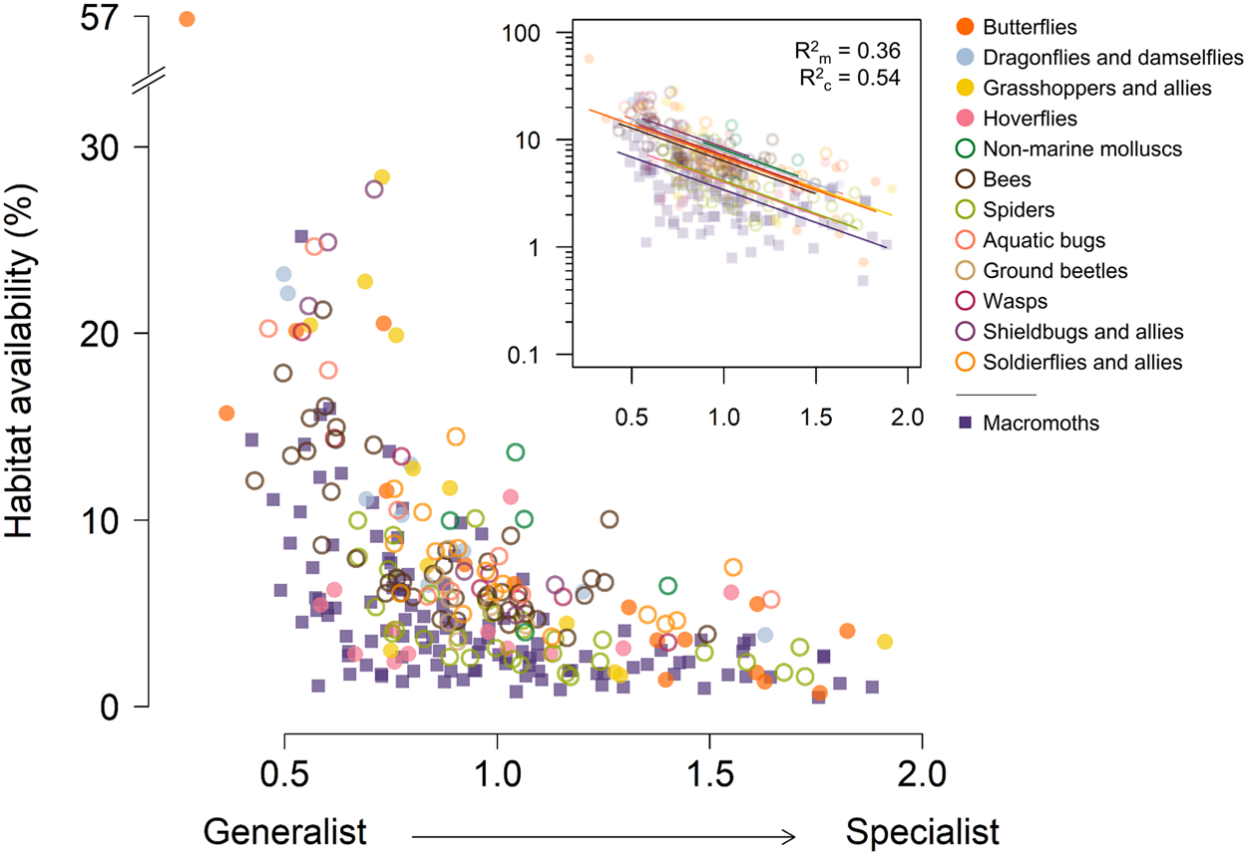
Relationship between habitat specialisation and habitat availability in species’ range margins. Plotted on untransformed axis and with log-linear scaling (inset, with fitted lines from a random intercept model). In the colour key, taxonomic groups are listed in descending order of geographic coverage of citizen-science recording: solid circles show four groups with high recording intensity, open circles show eight groups with lower recording intensity, squares show macromoths (different recording method).

For each of the 291 species we quantified habitat availability in their 1976–1990 range-margin landscapes, and a related measure of habitat specialisation^18^ – defined as the coefficient of variation (SD/mean) in the probability of occurrence across 18 satellite-derived vegetation or habitat classes^19^ (mapped at 1-ha resolution). While habitat specialisation and habitat availability are clearly related to one another, they are not interchangeable (Fig. 2). As for range shifts, variation in habitat specialisation and habitat availability are mainly features of differences between individual species, rather than between taxonomic groups (Fig. 1 and Tables S3–S5).

**Figure 3:**
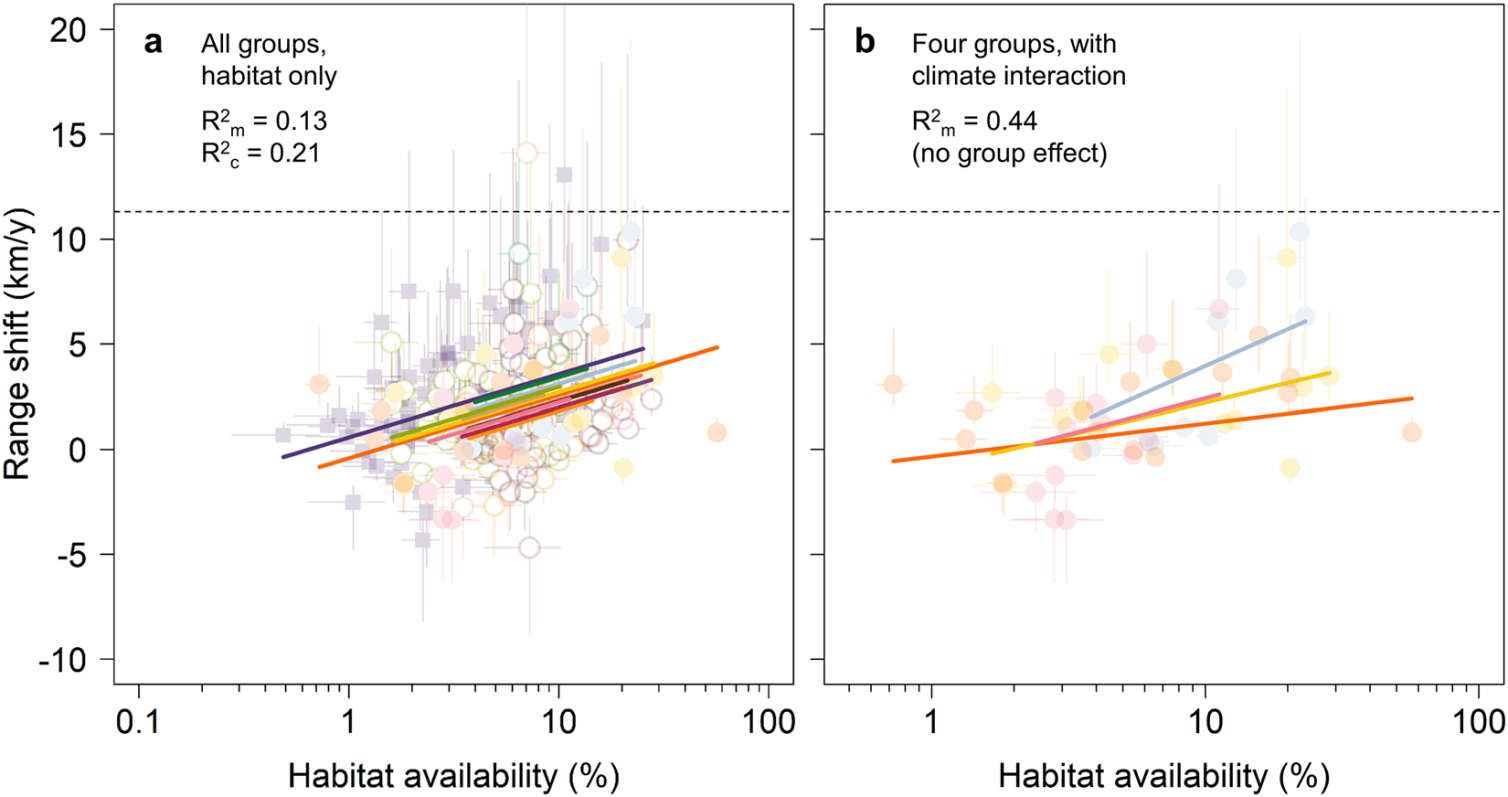
Relationships between habitat availability and species’ range shifts. (**a**) Random intercept model (conditional on group), including all 291 species across 13 taxonomic groups. (**b**) Habitat-climate interaction model, restricted to four groups with high recording intensity (butterflies, dragonflies and damselflies, grasshoppers and allies, and hoverflies; n = 49 species). In a, coloured lines show the random effect of taxonomic group on the fixed effect of log_10_-habitat availability in species’ range margins. In b, group has no effect but for illustration we plot the mean exposure to climate change for species within each taxonomic group. Error bars represent 95% CI around rates of range shift (given the breadth of recording periods) and habitat availability (from logistic regression). Dotted lines show the shift in mean annual isotherms given observed warming between the two recording periods (1976–1990 and 2001–2015; 11.2 km y^−1^, Fig. S1).

Habitat availability ranged from 0.5% of the range-margin landscape for the Sand Dart moth *Agrotis ripae* (restricted to coastal strandlines) to 57% for the Gatekeeper butterfly *Pyronia tithonus*. In a linear mixed-effects model, the marginal effect of habitat availability (R^2^_m_, which controls for all other covariates in the model) explains 13% of the observed variation in species’ range shifts, with an additional 8% conditional on group-level intercepts (R^2^_c_ = 21%; Fig. 3a). When data are restricted to the most reliably-recorded taxonomic groups (n = 49 species in four groups: butterflies, dragonflies and damselflies, grasshoppers and allies, and hoverflies) the marginal R^2^ increases to 22%, with no effect of group (Table S6).

Habitat specialisation, independent of landscape context, explains less of the variation. Levels of specialisation ranged from 0.3 for *P*. *tithonus* butterfly (habitat generalist) to 1.9 for the Bog Bush Cricket *Metrioptera brachyptera* (a wetland specialist). Relatively generalist species expanded polewards faster than more specialised species (Satterthwaite’s t-test: P = 0.0011, n = 291 species), explaining R^2^_m_ = 4% and R^2^_c_ = 6% of the observed variation (R^2^_m_ = 4% and R^2^_c_ = 11% for the most reliably-recorded groups). Thus, regardless of group-level differences in recording intensity, accounting for the differential expression of species’ habitat associations in different landscapes (i.e., comparing habitat availability *vs*. specialisation *per se* as predictor variables) substantially increases the variation in range shift that can be explained (Table S6).

### Interaction with climate

Some of the remaining variation between species may be due to species-specific sensitivities to different elements of the climate, and hence their exposure to climate change. In our calculations of habitat associations, we controlled for spatial differences in climate using annual 1-ha resolution maps of minimum temperature, accumulated warmth (degree-days above 5 °C), and moisture balance (ratio of rainfall to potential evapotranspiration)^20,21^. Hence, we defined exposure to climate change as the logged ratio of change in the climatic suitability of species’ range-margin landscapes, given the change in the average climate between 1976–1990 and 2001–2015 for these three climate variables.

**Figure 4:**
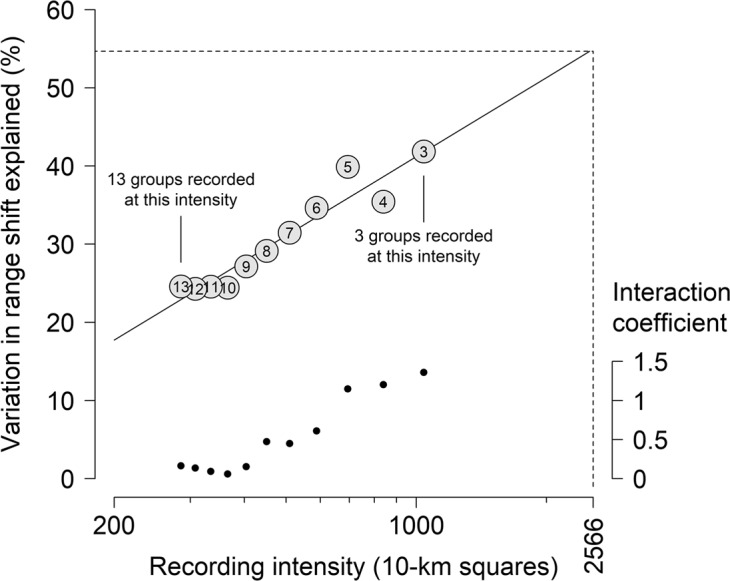
Relationship between recording intensity and the variation in range shift explained by habitat availability and climate. The fixed effects are log_10_-habitat availability at the range margin, exposure to climate change at the range margin, and their interaction. The higher the recording intensity, the steeper the interaction slope (black dots) and the greater the marginal R^2^ of habitat and climate (grey circles), up to a theoretical maximum of R^2^_m_ = 55% (dashed lines). Recording intensity is the number of 10 km × 10 km squares where at least 25% of the regional species richness for a taxonomic group was recorded in both 1976–1990 and 2001–2015 (mean across groups, up to a maximum 2566 squares in Britain). The pool of groups decreases from left to right as recording intensity increases (model degrees of freedom fixed by drawing 30 species from any three qualifying groups, plotting mean values over 10,000 repetitions).

We found that while exposure to climate change is positively associated with rates of range shift (Satterthwaite’s t-test: P = 0.00048, n = 291 species), this explains less variation than habitat availability (R^2^_m_ = 4% and R^2^_c_ = 4%, increasing to R^2^_m_ = 16% and R^2^_c_ = 19% for the most reliably-recorded groups; Table S6). We found support for models including both habitat availability and exposure to climate change (ΔcAIC = 2 compared with habitat-only model^22^) and for their statistical interaction, especially for the most reliably-recorded groups (ΔcAIC = 14). Thus, species exposed to the greatest increases in climatic suitability at their range margins have expanded polewards fastest, but only to the extent that habitat was available (R^2^_m_ = 44% with no effect of group; Satterthwaite’s t-test on interaction term: P = 0.00069, n = 49 species; Fig. 3b and Table S6). Ranking groups by the geographic coverage of biological recording across both time periods, we found that the slope of the interaction term (and variation explained by the model) increases predictably with recording intensity (Pearson correlation coefficient, r = 0.96). Extrapolating to assume universal geographic coverage of recording for all study taxa implies that habitat availability and its interaction with climate could explain over half of the observed variation in species’ range shifts (Fig. 4).

## Discussion

Our analysis confirms that range-margin dynamics vary greatly among species, and finds that up to a quarter of this variation (depending on recording effort) can be explained independently of species-level differences in exposure to climate change, by the interplay between species’ habitat associations and the landscapes they encounter during range expansion. This is likely to be a minimum estimate of the effect of habitat, given that satellite-derived habitat classes do not provide a full species-eye view of environmental suitability (including available resources), land cover may change over time, and evolutionary changes in resource use can take place during range expansion^20,23–28^.

Around half of the variation in range shifts can be explained when also accounting for species’ differential exposure to climate change (controlling for recording effort and including habitat-climate interactions). On their own, species-level differences in climate sensitivity and exposure explain comparatively little of the variation in range shifts, which is perhaps surprising given the likely overall role of climate change in driving mean polewards expansions. The interaction with habitat availability, however, suggests strong climate forcing masked by habitat constraints. For example, the Emperor dragonfly *Anax imperator* has expanded polewards at 10 km y^−1^ in response to a 43% improvement in climatic suitability, facilitated by ample range-margin habitat (22% of the landscape); conversely, the Scarce Chaser dragonfly *Libellula fulva* has been unable to respond quickly (40 m y^−1^) across a landscape with 4% habitat availability despite a similar improvement in range-margin climate (Table S7). The strength of the habitat-climate interaction term increases log-linearly with the quality of the species data, suggesting that shallower interaction slopes among less well-recorded taxa are a matter of reduced information rather than a reduction in actual importance.

The habitat-climate interaction emerges from multiple underlying interactions between the physical environment in each habitat class, the biological interactions found within it (including with resources and natural enemies), and microclimatic conditions generated by interactions between the vegetation and broader-scale climate^29,30^. More localised differences within each habitat class will also arise because of these multiple interactions, affecting spatial and temporal patterns of population growth rate (affecting the likelihood of population establishment), densities (affecting propagule numbers) and individual behavioural responses (affecting dispersal rates)^31,32^. Here, to include as many species and landscapes as possible, we did not attempt to isolate any particular mechanism of habitat association or climate interaction: we took a resource-based view of habitat^33^, recognising that a species occupies particular habitat classes because certain resources (e.g., host plants, prey, mutualists), structural elements (e.g., that enable spider webs to be built), or micro-environments (e.g., sheltered microclimates) are present somewhere within that class, and/or because negative influences (e.g., natural enemies, disruptive land management) are absent. For example, hedgerow species can be positively associated with an ‘arable’ habitat class (albeit with low habitat availability), which is a true reflection of where many of these species live, given that field boundaries are commonly demarcated by hedgerows, and that such linear features are nested within the 20–30 m grain size of satellite imagery^34^. Thus, the full habitat surface available to any species is finer-grained than depicted here and would likely explain an even higher percentage of the variation among species.

Our findings demonstrate the ubiquitous constraint that habitat has already imposed on climate-driven range shifts across multiple taxonomic groups. Given high uncertainty in future levels of climate warming, combined with even greater uncertainty around the impact of warming on biologically-relevant weather patterns^21,35^, the fact that habitat alone explains a large proportion of the variation in species’ responses is important for planning and adaptation. Global repositories of species’ distribution data and remotely-sensed habitat information make discerning habitat associations and range-margin conditions achievable for millions of species. Predictions of climate-change impact, whether based on species’ traits or their climate exposure^21^ should, wherever possible, include this information, to better foresee and ameliorate landscape resistance to species’ movements under climate change.

## Methods

### Study region

The study region encompassed 2566 Ordnance Survey 10 km × 10 km grid squares (hectads) covering the British mainland plus any near-shore islands connected to the mainland by the contiguous spread of hectads. During the first recording period (1976–1990), the mean annual temperature was 8.5 °C, increasing to 9.3 °C during the second recording period (2001–2015)^36^. The level of warming was therefore 0.8 °C (0.03 °C y^−1^) across the 25-year interval between the midpoints of the two recording periods. Given the latitudinal gradient in mean annual temperature, this equates to a 257–293 km northward shift in isotherms, depending on latitude (Fig. S1). At the median 1976–1990 range margin for our study species, the northward shift in isotherms was 281 km (11.2 km y^−1^).

### Species records

We considered all animal groups represented in the UK Biological Records Centre (BRC, www.brc.ac.uk) and included any group that contained at least five species meeting our inclusion criteria (see sections below). We identified 13 taxonomic groups, all invertebrates, with sufficient data for inclusion (Table S1), including carnivores, herbivores and omnivores, aquatic (freshwater) and terrestrial taxa, groups that disperse in the soil, by walking, by ballooning and by active flight, and spanning orders of magnitude in body mass.

Each group was covered by a formal recording scheme (Table S1). The majority of species records were collected by citizen scientist recorders, before being collated and cleaned by experts in the group/region to filter out possible errors. We retained the taxonomic distinctions and groupings used by these recording schemes (e.g., butterflies and macromoths were treated as separate groups, whereas dragonflies and damselflies were aggregated). Therefore, ‘group effects’ may reflect differences in the recording schemes as well as the effects of taxonomic group *per se*.

Each biological record represents a unique location × date observation of species presence. We removed records with ambiguous taxonomy (*sensu lato*, *sensu auct*, naming multiple species or identified only to genus). Species listed with a sub-species trinomial, with the label *sensu stricto*, with variants or different morphs were aggregated at the species level. When analysing range shifts, we used all records with at least hectad-level spatial accuracy that could be unambiguously assigned to one of the two recording periods (1976–1990 and 2001–2015). For habitat and climate associations, we used day-specific records accurate to 1-ha resolution across the period 1976–2015 (for the 291 species included in the final analysis, 74% of records had this level of spatial and temporal precision, ranging from 55% in the first recording period to 80% in the second recording period).

### Criteria for species inclusion

We selected non-migratory species that reach their northern (cool) range boundaries in southern/lowland Britain. We defined these species as having 90% of their 1976–1990 distribution in the warmest 50% of the study region^36^ (Fig. S1), with none of these records within 100 km of the north coast. Since these species have historically been concentrated in the warmer half of Britain, it is reasonable to postulate that they might be favoured by climate warming, and that their distributions would be predicted to expand (generally polewards). As non-migrants, the range extensions we document represent the establishment of new populations over multiple generations.

We excluded species classified as non-native, alien-native hybrid, unknown origin, and those that are dependent on non-native species, as defined by the BRC and the GB Non-native Species Information Portal^37^. We also excluded vagrants and species thought to be extinct from the study region, including species that have been reintroduced following extinction (e.g., Large Blue butterfly *Maculinea arion*).

This resulted in an eligible species set of 1570 species in 28 groups. Of these, 421 species had sufficient data to calculate range shifts, of which 305 (17 groups) also had sufficient data to calculate habitat associations (criteria detailed below). Excluding groups with fewer than five species resulted in a final dataset of 291 species in 13 groups.

### Range-shift calculations

To calculate range shifts, we first controlled for changes in recorder effort over time (1976–1990 to 2001–2015). We restricted distribution data to ‘well-recorded’ hectads, for which at least 10% of the regional species pool for a group was recorded present in both recording periods^38^ (Fig. S2). For each group × hectad, we defined the regional species pool as the total number of species recorded in the nearest 100 hectads^17^, using all species in the database for a given taxonomic group (i.e., regardless of the above inclusion criteria).

For all species occupying at least 20 such hectads in both recording periods, we calculated northern (cool) range margins as the mean latitude of the ten-northernmost occupied hectads. We checked that species had sufficient area to expand or retreat from their 1976–1990 range margins. Hence, we excluded any species with fewer than ten recorded (as above) hectads within 100 km to the north of the range margin, and ten such hectads within 100 km to the south of the range margin^17^. For the remaining species, we defined range shifts as the latitudinal change (km) in range margins between 1976–1990 and 2001–2015. We converted latitudinal changes to annual rates (km y^−1^) by sampling random dates (10,000 times) from within each of the two 15-year recording periods, dividing latitudinal shifts by each sampled interval, and recording the median rate for each species. When calculating summary rates of range shift across multiple species (e.g., Fig. 1 and Table S2), we did this separately for each sampled time interval and report the 95% confidence interval around the sample median.

### Habitat classification

To identify habitat classes, we used a 25 m × 25 m land-cover map for Britain (LCM2007). This map was created by the NERC Centre for Ecology and Hydrology^19^, using combined summer and winter satellite data (Landsat-TM5, IRS-LISS3, SPOT-4 and SPOT-5 sensors, pixel size of 20–30 m) enhanced with cartographical information (e.g., Ordnance Survey data, soil types, agricultural census boundaries and urban extents). The classification was trained and validated using a large network of habitat surveys and ground reference points, producing an overall accuracy of 83%. Out of 23 habitat classes identified in LCM2007, we discarded one (saltwater), retained 14 as originally mapped, and created four aggregate classes from the remaining eight: ‘heather’ and ‘heather grassland’ became ‘dwarf shrub heath’; ‘supra-littoral rock’ and ‘littoral rock’ became ‘coastal rock’; ‘supra-littoral sediment’ and ‘littoral sediment’ became ‘coastal sediment’; ‘suburban’ and ‘urban’ became ‘built-up and gardens’.

### Climate estimates

We estimated climatic conditions corresponding to each 1-ha species record, and the wider range-margin landscape, by spatially downscaling 5 km × 5 km resolution UKCP09 climate grids provided by the UK Met Office^36^. For each month of the study period (1976–2015), we used universal kriging with linear dependence on elevation to spatially interpolate mean daily minimum (T_Min_) and mean daily maximum (T_Max_) air temperatures to 1-ha resolution (Fig. S3). Elevation data were from the Ordnance Survey Terrain 50 product, resampled to the 1-ha grid. For each month and climate variable, we constructed spherical and exponential variograms with distance cut-offs of 100 km and retained whichever had the lowest sum of squared errors. Kriging was implemented using the nearest 30 points. Using the same procedure, but with no dependence on elevation, we kriged UKCP09 monthly rainfall and sunshine-hours grids. Sunshine hours were then converted to sunshine fraction (dividing by day length) to estimate the proportion of the day was typically cloud-free in a given month.

Using sunshine fraction and information on topographic position, we adjusted maximum temperatures for solar radiation. First, we used the Solar Area Radiation tool in ESRI ArcMap to calculate solar radiation at 1-ha resolution, once assuming a ‘flat’ surface (SR_Flat_) and then again accounting for the influence of slope, aspect and hill shading (SR_Topo_). In each case, sky conditions were weighted linearly towards clear skies (transmissivity = 0.7, diffuse fraction = 0.2) or overcast conditions (transmissivity = 0.2, diffuse fraction = 0.7) depending on the sunshine fraction for the corresponding month. We then used the ratio of SR_Topo_ and SR_Flat_ to scale each grid cell’s diurnal temperature range^39^, as follows:

$${{\rm{T}}}_{{\rm{MaxSR}}}={{\rm{T}}}_{{\rm{Min}}}+{{\rm{SR}}}_{{\rm{Topo}}}/{{\rm{SR}}}_{{\rm{Flat}}}\times ({{\rm{T}}}_{{\rm{Max}}}-{{\rm{T}}}_{{\rm{Min}}}).$$

Mean daily temperatures were calculated as the mean of daily minima and daily maxima. Finally, we derived three biologically-relevant annual climate variables^21^: minimum winter temperature, degree-days above 5 °C, and the ratio of annual rainfall to potential evapotranspiration^40^. We defined annual variables over 12-month periods beginning 1 September, because this represents a more biologically-relevant annual cycle in Britain than a calendar year (which begins part-way through winter)^35^.

### Habitat and climate associations

We identified habitat and climate associations using quasibinomial regression of species presence or absence overlaid on the 18 habitat classes (categorical predictor) and the three annual climate variables (continuous predictors)^20^. The regression equation for each species was used to estimate its probability of occurrence in each habitat class, under the assumption of equal availability of all habitat classes (i.e., as close as is possible to a ‘species characteristic’) and with climate fixed at mean (centred) values. We defined levels of habitat specialisation to be the coefficient of variation across these 18 probabilities^18^, producing a species’ specialisation score which, for our dataset, ranged from 0.3 (generalist) to 1.9 (specialist). Results are summarised by taxonomic group in Table S3 and reported for individual species in Table S7.

Given the finer grain of the land-cover map (25 m × 25 m) compared with the species data (1 ha), some species records could potentially be associated with multiple habitat classes. In these cases, we assigned the majority habitat class for the 1-ha pixel, and included weights to indicate the proportion of the pixel covered by this habitat^41^. Climate values were specific to the year of a species observation, except where a species was observed in the same 1-ha pixel in multiple years, in which cases we assigned the mean climate values across those years.

We otherwise took all recorded presences to be ‘true’ for the purposes of modelling and included in the final analysis all species with presence records in at least 200 distinct 1-ha pixels (approximately 20 presence pixels for each parameter estimated by the model; mean records [distinct pixels] per species = 8,167 [2,238], median = 1,390 [694], range = 260–343,040 [201–104,960]). Inferring absence data from presence-only datasets is inherently more difficult. To minimise the number of false absences, we applied the following controls. First, we only included as potential absences those pixels that had been visited by recorders of the same recording scheme (as deduced from records of other species within the same recording scheme), within 50-km of a presence observation for the target species, and excluding landscapes occupied during only one recording period. We did this to account for, respectively, lack of visitation by appropriate recorders, historical barriers to dispersal, and changes in land cover.

Second, we filtered absences according to time of year, given their location, for example to avoid treating late summer data as absences if the target species’ flight period is in spring. We did this by fitting smooth phenology curves to the frequencies of record dates (days of the year) for the target species. To account for spatial variations in phenology, we restricted these records to within 50 km of a particular absence site. Potential absences with record dates in the tails (lower or upper 10% area under the curve) of these location- and species-specific phenology curves were excluded. In the few cases where smoothing splines could not be constructed, we defined the tails of the phenology curve directly from the raw dates (10^th^ and 90^th^ percentiles, correcting for year-breaks where needed).

The remaining absences were from 1-ha pixels visited under the same recording scheme as the target species, in landscapes near where the target species had been recorded in both time periods, and within the appropriate phenological time windows. The absences still varied in reliability, however, because some qualifying pixels had only been visited only once, whereas others had been visited multiple times. Third, therefore, we weighted absence data by the probability of recording the target species if it was present, given the number times (*t*) the absence pixel was visited:

$$\frac{1}{n}\sum _{s=\mathrm{1..}n}1-{(1-{p}_{s})}^{t}$$

That is, one minus the probability of failing to detect the species on every occasion, where the *p*_*s*_ are probabilities of detection across *n* known presence sites for the target species (calculated as the number of times the species was recorded in site *s* divided by the number of times *s* was visited). To account for spatial variations in abundance (and therefore detectability) we calculated *p*_*s*_ using data restricted to within 50 km of the absence site.

### Conditions at species’ range margins

We obtained spatial estimates of habitat availability by predicting each species’ regression model back on the land-cover map, with climate fixed at mean (centred) values. Habitat availability at the range margin was defined as the mean value across all 25 m × 25 m land-cover pixels in a circular 50-km buffer around the ten-northernmost hectads (or >10 hectads when >1 hectad tied for having the 10^th^ highest latitude) that were used to define the range margin in the first recording period (1976–1990); i.e., landscapes across which the species had potential to expand or retract over time. Habitat availability for individual species ranged from 0.5% (very little of the landscape could be colonised) to 57% of the landscape (ample opportunity for expansion; Tables S4 and S7). Habitat availability exhibited positive skew (Fig. 1; Shapiro-Wilk, W = 0.75, P < 10^−20^, n = 291 species) and so we log_10_-transformed these values to improve normality (W = 0.997, P = 0.90).

We defined exposure to climate change as the logged ratio of mean range-margin conditions in 1976–1990 versus 2001–2015. That is, we predicted each species’ regression model back on the land-cover map twice, first with annual climate set to its mean condition for the period 1976–1990, and second with climate set to its mean condition for 2001–2015. Since habitat data were constant in the model, the ratio of these predictions was >1 if climatic suitability improved over time, <1 if climatic suitability deteriorated, and =1 if there was no change in climatic suitability. We defined exposure using logged ratios so that there was symmetry about the no-change line; i.e., absolute exposure had the same magnitude when climate was improving as when it was deteriorating. Our usage of the term ‘exposure’ equates to change in climatic suitability for a species and encompasses both sensitivity to climate and the extent to which relevant aspects of climate have changed (*cf*. IPCC terminology of exposure being independent of sensitivity^21^).

### Models of range shift

We modelled species’ observed range shifts as linear functions of habitat specialisation, log_10_-transformed habitat availability at the range margin, and exposure to climate change at the range margin. We constructed single-predictor models, as well as multivariate models where collinearity among predictors was low (Table S6): habitat specialisation and log_10_-habitat availability were highly correlated (r = −0.53; Fig. 2) and so we did not include both in the same model, whereas exposure to climate change was uncorrelated with both of these predictors (r = −0.02 and r = 0.07, respectively). To account for phylogenetic relatedness and methodological differences in recording between taxonomic groups (i.e., across recording schemes), we used linear mixed-effects models with taxonomic group as the random intercept term (Table S6).

### Sensitivity to recording level

We ranked the 13 taxonomic groups by descending geographic coverage of citizen-science recording, defined by the number of hectads where there has been sufficient recording for at least 25% of the regional species richness (considering the nearest 100 hectads) to have been sampled in both recording periods (Table S1). In Fig. 1, we summarise between-species variation separately for: (1) four groups with the high intensity recording; (2) eight groups with lower intensity recording; and (3) macromoths. We plotted macromoths separately because, unlike other groups, moth recording uses attractant methods (light traps at night) so that the areas sampled – and thus habitat associations – are more uncertain.

The proportion of variation in range shift that could be explained was higher for taxonomic groups with higher recording intensity, but the signs of the relationships were consistent (Table S6), demonstrating that the patterns we report are qualitatively robust to recording intensity. In Fig. 4 we systematically varied the threshold of recording coverage, above which species are included in the habitat-climate interaction model. For example, when the recording threshold is low, many groups are eligible for inclusion; when the threshold is high, only the best-recorded groups are included. For consistency in statistical power across different levels of group inclusion, each point in Fig. 4 was generated by averaging over 10,000 randomised draws of 30 species from three qualifying groups.

We conducted statistical and spatial analyses using R version 3.6.1, with packages lme4^42^, lmerTest^43^, MuMIn^44^, cAIC4^22^, doParallel^45^, raster^46^ and rgeos^47^.

## Supplementary information

Supplementary information

## Data Availability

The datasets that support this study are available from the following sources: species distribution data via the UK Biological Records Centre (www.brc.ac.uk); land-cover data under licence via EDINA (https://digimap.edina.ac.uk); climate data from the UK Met Office (https://catalogue.ceda.ac.uk/uuid/87f43af9d02e42f483351d79b3d6162a). Downscaled climate grids and R-scripts for our analysis are archived online at https://webfiles.york.ac.uk/CIARG/10.1038_s41598-019-51582-2.

## References

[1] Parmesan C, Yohe G (2003). A globally coherent fingerprint of climate change impacts across natural systems. Nature.

[2] Tingley MW, Monahan WB, Beissinger SR, Moritz C (2009). Birds track their Grinnellian niche through a century of climate change. Proc. Natl. Acad. Sci. USA.

[3] Scheffers, B. R. *et al*. The broad footprint of climate change from genes to biomes to people. *Science*. **354** (2016). 10.1126/science.aaf7671 27846577

[4] Lenoir J (2010). Going against the flow: potential mechanisms for unexpected downslope range shifts in a warming climate. Ecography..

[5] Angert AL (2011). Do species’ traits predict recent shifts at expanding range edges?. Ecol. Lett..

[6] Crimmins SM, Dobrowski SZ, Greenberg JA, Abatzoglou JT, Mynsberge AR (2011). Changes in climatic water balance drive downhill shifts in plant species’ optimum elevations. Science..

[7] MacLean, S. A. & Beissinger, S. R. Species’ traits as predictors of range shifts under contemporary climate change: A review and meta-analysis. *Glob*. *Chang*. *Biol*. 4094–4105 (2017). 10.1111/gcb.13736 28449200

[8] Freeman BG, Lee-Yaw JA, Sunday JM, Hargreaves AL (2018). Expanding, shifting and shrinking: The impact of global warming on species’ elevational distributions. Glob. Ecol. Biogeogr..

[9] Hill JK (2001). Impacts of landscape structure on butterfly range expansion. Ecol. Lett..

[10] Warren MS (2001). Rapid responses of British butterflies to opposing forces of climate and habitat change. Nature.

[11] Mair L (2014). Abundance changes and habitat availability drive species’ responses to climate change. Nat. Clim. Chang..

[12] Carroll MJ (2015). Hydrologically driven ecosystem processes determine the distribution and persistence of ecosystem-specialist predators under climate change. Nat. Commun..

[13] Liang Y, Duveneck MJ, Gustafson EJ, Serra-Diaz JM, Thompson JR (2018). How disturbance, competition, and dispersal interact to prevent tree range boundaries from keeping pace with climate change. Glob. Chang. Biol..

[14] Årevall J, Early R, Estrada A, Wennergren U, Eklöf AC (2018). Conditions for successful range shifts under climate change: The role of species dispersal and landscape configuration. Divers. Distrib..

[15] Macgregor, C. J. *et al*. Climate-induced phenology shifts linked to range expansions in species with multiple reproductive cycles per year. *Nat*. *Commun*. In press (2019). 10.1038/s41467-019-12479-w PMC6813360 31649267

[16] Chen I, Hill JK, Ohlemüller R, Roy DB, Thomas CD (2011). Rapid range shifts of species associated with high levels of climate warming. Science..

[17] Mason SC (2015). Geographical range margins of many taxonomic groups continue to shift polewards. Biol. J. Linn. Soc..

[18] Julliard R, Clavel J, Devictor V, Jiguet F, Couvet D (2006). Spatial segregation of specialists and generalists in bird communities. Ecol. Lett..

[19] Morton, D. *et al*. *Final Report for LCM2007—The New UK Land Cover Map*. *CEH Project Number: C03259; Countryside Survey Technical Report No*.*11/07*. (NERC/Centre for Ecology & Hydrology, 2011).

[20] Pateman RM, Thomas CD, Hayward SAL, Hill JK (2016). Macro- and microclimatic interactions can drive variation in species’ habitat associations. Glob. Chang. Biol..

[21] Foden WB (2019). Climate change vulnerability assessment of species. Wiley Interdiscip. Rev. Clim. Chang..

[22] Säfken B, Rügamer D, Kneib T, Greven S (2018). Conditional model selection in mixed-effects models with cAIC4. arXiv.

[23] Weiss-Lehman C, Hufbauer RA, Melbourne BA (2017). Rapid trait evolution drives increased speed and variance in experimental range expansions. Nat. Commun..

[24] Oliver T, Hill JK, Thomas CD, Brereton T, Roy DB (2009). Changes in habitat specificity of species at their climatic range boundaries. Ecol. Lett..

[25] Oliver TH, Thomas CD, Hill JK, Brereton T, Roy DB (2012). Habitat associations of thermophilous butterflies are reduced despite climatic warming. Glob. Chang. Biol..

[26] Thomas CD (2001). Ecological and evolutionary processes at expanding range margins. Nature.

[27] Hanski I, Mononen T (2011). Eco-evolutionary dynamics of dispersal in spatially heterogeneous environments. Ecol. Lett..

[28] Pateman RM, Hill JK, Roy DB, Fox R, Thomas CD (2012). Temperature-dependent alterations in host use drive rapid range expansion in a butterfly. Science..

[29] De Frenne P (2013). Microclimate moderates plant responses to macroclimate warming. Proc. Natl. Acad. Sci. USA.

[30] Suggitt AJ (2018). Extinction risk from climate change is reduced by microclimatic buffering. Nat. Clim. Chang..

[31] Wilson RJ, Davies ZG, Thomas CD (2009). Modelling the effect of habitat fragmentation on range expansion in a butterfly. Proc. R. Soc. Ser. B.

[32] Hodgson JA, Thomas CD, Dytham C, Travis JMJ, Cornell SJ (2012). The speed of range shifts in fragmented landscapes. PLoS One.

[33] Dennis, R. L. H. *A resource-based habitat view for conservation: butterflies in the British Landscape*. (Wiley-Blackwell, 2010).

[34] Sullivan MJP (2017). A national-scale model of linear features improves predictions of farmland biodiversity. J. Appl. Ecol..

[35] Palmer G (2017). Climate change, climatic variation and extreme biological responses. Philos. Trans. R. Soc. Ser. B.

[36] Hollis, D. & McCarthy, M. UKCP09: Met Office gridded and regional land surface climate observation datasets. *UK Met Office*, *Centre for Environmental Data Analysis* Available at, http://catalogue.ceda.ac.uk/uuid/87f43af9d02e42f483351d79b3d6162a (2017).

[37] Roy HE (2014). GB Non-native Species Information Portal: documenting the arrival of non-native species in Britain. Biol. Invasions.

[38] Hickling R, Roy DB, Hill JK, Fox R, Thomas CD (2006). The distributions of a wide range of taxonomic groups are expanding polewards. Glob. Chang. Biol..

[39] Bennie J, Huntley B, Wiltshire A, Hill MO, Baxter R (2008). Slope, aspect and climate: Spatially explicit and implicit models of topographic microclimate in chalk grassland. Ecol. Modell..

[40] Hargreaves GH, Allen RG (2003). History and evaluation of Hargreaves evapotranspiration equation. J. Irrig. Drain. Eng..

[41] Chetcuti, J., Kunin, W. E. & Bullock, J. M. A weighting method to improve habitat association analysis: tested on British carabids. *Ecography*. 1395–1404 (2019).

[42] Bates D, Mächler M, Bolker B, Walker S (2014). Fitting linear mixed-effects models using lme4. J. Stat. Softw..

[43] Kuznetsova A, Brockhoff PB, Christensen RH (2017). B. lmerTest Package: tests in linear mixed effects models. J. Stat. Softw..

[44] Barton, K. MuMIn: Multi-Model Inference. R package version 1.43.6 (2019).

[45] Microsoft-Corporation & Weston, S. doParallel: Foreach Parallel Adaptor for the ‘parallel’ Package. R package version 1.0.11 (2017).

[46] Hijmans R (2017). J. raster: Geographic Data Analysis and Modeling. R package version.

[47] Bivand R, Rundel C (2018). rgeos: Interface to Geometry Engine - Open Source (‘GEOS’). R package version.

