# Supplementary material for: Habitat availability explains variation in climate-driven range shifts across multiple taxonomic groups
Source: Sci Rep. 2019 Oct 21;9:15039. doi: 10.1038/s41598-019-51582-2 (PMC6803766; doi:10.1038/s41598-019-51582-2)
Supplement: Supplementary file 1 — Supplementary information [file 41598_2019_51582_MOESM1_ESM.pdf]

# Supplementary Information

## Habitat availability explains variation in climate-driven range shifts across multiple taxonomic groups

Philip J. Platts, Suzanna C. Mason, Georgina Palmer, Jane K. Hill, Tom H. Oliver, Gary D. Powney, Richard Fox, Chris D. Thomas

**Table S1.** Details of citizen-scientist recording schemes. Data were obtained on 02 June 2017 from the UK Biological Records Centre (www.brc.ac.uk/theme/datasets), and represent opportunistic point observations at or near breeding sites (but see Remarks). Recording level is the number of hectads (10 km × 10 km grid squares, out of a possible 2566 in the study area) where at least 10% or 25% of the regional species pool was sampled in both recording periods (1976-1990 and 2001-2015).

| Taxonomic group | Recording scheme name and affiliation | National organiser(s) | Remarks | Number of species | Recording level (10%) | Recording level (25%) |
|---|---|---|---|---|---|---|
| 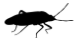 Aquatic bugs[1] | Aquatic Heteroptera Recording Scheme (Aquatic Coleoptera Conservation Trust) | Dr Garth Foster | The Trust is a Registered Charity No. SCO37556 | 9 | 166 | 35 |
| 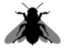 Bees[2] | Bees, Wasps and Ants Recording Scheme / Society | Mr Mike Edwards | Thanks to the committee and members of BWARS for permission to use their data | 43 | 164 | 39 |
| 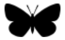 Butterflies | Butterflies for the New Millennium (Butterfly Conservation, BC) | Mr Richard Fox | Includes long-term monitoring transects. BC is a Registered Charity No. 254937 and No. SCO39268 | 16 | 2157 | 1710 |
| 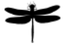 Dragonflies and damselflies[3] | Dragonfly Recording Network (British Dragonfly Society)[4] | Mr David Hepper | Registered charity No. 1168300 | 9 | 1350 | 813 |
| 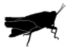 Grasshoppers and allies | Grasshoppers and Related Insects Recording Scheme of Britain and Ireland | Prof Peter Sutton<br>Dr Björn Beckmann | Both visual and acoustic identification | 12 | 831 | 234 |
| 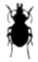 Ground beetles[4] | Ground Beetle Recording Scheme | Dr Mark Telfer | – | 8 | 182 | 23 |
| 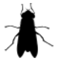 Hoverflies[3] | Hoverfly Recording Scheme (Dipterists Forum) | Dr Roger Morris<br>Dr Stuart Ball | – | 12 | 540 | 116 |

| Taxonomic group | | Recording scheme name and affiliation | National organiser(s) | Remarks | Number of species | Recording level (10%) | Recording level (25%) |
|---|---|---|---|---|---|---|---|
| 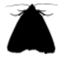 | Macromoths[3] | National Moth Recording Scheme (Butterfly Conservation, BC) | Mr Richard Fox | Includes use of light traps at night. BC is a Registered Charity No. 254937 and No. SCO39268 | 116 | 1034 | 600 |
| 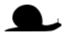 | Non-marine molluscs | Non-marine Mollusc Recording Scheme (Conchological Society of Great Britain and Ireland) | Mr Adrian Norris | The Society is a Registered Charity No. 208205 | 5 | 243 | 54 |
| 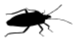 | Shieldbugs and allies[1] | Terrestrial Heteroptera Recording Scheme | Dr Tristan Bantock | – | 6 | 134 | 17 |
| 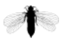 | Soldierflies and allies | Soldierflies and Allies Recording Scheme (Dipterists Forum) | Mr Martin Harvey | Additional records obtained direct from recording scheme (28.06.2017) | 16 | 182 | 15 |
| 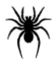 | Spiders | Spider Recording Scheme (British Arachnological Society) | Mr Peter Harvey | The Society is a Registered Charity No. 260346 and No. SC044090 | 31 | 242 | 38 |
| 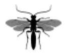 | Wasps[2] | Bees, Wasps and Ants Recording Scheme / Society | Mr Mike Edwards | Thanks to the committee and members of BWARS for permission to use their data | 8 | 94 | 18 |

Group icons are from http://phylopic.org/: under CC0 1.0 (https://creativecommons.org/publicdomain/zero/1.0/), except where superscripted in which case CC BY 3.0 (https://creativecommons.org/licenses/by/3.0/) with credits to [1]Dave Angelini, [2]Melissa Broussard, [3]Gareth Monger, [4][T. Michael Keesey (vectorization), Thorsten Assmann, Jörn Buse, Claudia Drees, Ariel-Leib-Leonid Friedman, Tal Levanony, Andrea Matern, Anika Timm, and David W. Wrase (photography)].

**Table S2.** Summary statistics for species' range shifts (km $y^{-1}$), measured between 1976-1990 to 2001-2015. Positive values indicate northward (poleward) expansion, and negative values indicate southward retreat.

| Taxonomic group | Mean | Median | IQR | Min | Max |
|---|---|---|---|---|---|
| Aquatic bugs | 2.02 | 1.64 | 2.63 | -2.16 | 5.95 |
| Bees | 1.86 | 1.32 | 2.87 | -2.04 | 9.94 |
| Butterflies | 1.80 | 1.84 | 2.89 | -1.60 | 5.39 |
| Dragonflies and damselflies | 3.70 | 1.04 | 5.71 | 0.04 | 10.34 |
| Grasshoppers and allies | 2.49 | 2.28 | 2.20 | -1.72 | 9.10 |
| Ground beetles | 2.32 | 0.96 | 1.86 | -2.75 | 14.09 |
| Hoverflies | 0.73 | 0.70 | 3.66 | -3.35 | 6.67 |
| Macromoths | 2.33 | 1.74 | 2.84 | -4.31 | 13.05 |
| Non-marine molluscs | 4.92 | 4.55 | 5.63 | 0.88 | 9.30 |
| Shieldbugs and allies | 1.12 | 1.68 | 2.58 | -4.67 | 4.07 |
| Soldierflies and allies | 0.82 | 0.64 | 1.93 | -2.67 | 3.99 |
| Spiders | 1.79 | 1.88 | 2.14 | -1.40 | 7.39 |
| Wasps | 0.92 | 0.98 | 1.69 | -1.04 | 2.67 |
| All groups | 2.05 | 1.64 | 2.79 | -4.67 | 14.09 |

**Table S3.** Summary statistics for species' specialisation indices. High values indicate a high level of habitat specialisation.

| Taxonomic group | Mean | Median | IQR | Min | Max |
|---|---|---|---|---|---|
| Aquatic bugs | 0.87 | 0.84 | 0.40 | 0.46 | 1.64 |
| Bees | 0.87 | 0.88 | 0.38 | 0.43 | 1.49 |
| Butterflies | 1.16 | 1.34 | 0.87 | 0.27 | 1.82 |
| Dragonflies and damselflies | 0.87 | 0.80 | 0.23 | 0.50 | 1.63 |
| Grasshoppers and allies | 0.97 | 0.82 | 0.45 | 0.56 | 1.91 |
| Ground beetles | 0.92 | 0.89 | 0.04 | 0.86 | 1.07 |
| Hoverflies | 0.93 | 0.88 | 0.32 | 0.58 | 1.55 |
| Macromoths | 0.96 | 0.88 | 0.39 | 0.42 | 1.88 |
| Non-marine molluscs | 1.09 | 1.06 | 0.02 | 0.89 | 1.40 |
| Shieldbugs and allies | 0.83 | 0.82 | 0.38 | 0.56 | 1.14 |
| Soldierflies and allies | 1.03 | 0.95 | 0.34 | 0.76 | 1.56 |
| Spiders | 1.06 | 1.00 | 0.33 | 0.67 | 1.72 |
| Wasps | 0.93 | 0.97 | 0.34 | 0.54 | 1.40 |
| All groups | 0.96 | 0.91 | 0.37 | 0.27 | 1.91 |

**Table S4.** Summary statistics for habitat availability in species' 1976-1990 range-margin landscapes (%).

| Taxonomic group | Mean | Median | IQR | Min | Max |
|---|---|---|---|---|---|
| Aquatic bugs | 11.7 | 8.1 | 12.0 | 5.7 | 24.6 |
| Bees | 8.5 | 6.7 | 4.9 | 3.7 | 21.3 |
| Butterflies | 10.4 | 5.4 | 9.5 | 0.7 | 56.9 |
| Dragonflies and damselflies | 11.6 | 10.3 | 6.5 | 3.8 | 23.2 |
| Grasshoppers and allies | 11.5 | 9.6 | 16.7 | 1.7 | 28.4 |
| Ground beetles | 5.7 | 5.6 | 2.1 | 3.5 | 8.5 |
| Hoverflies | 4.5 | 3.6 | 2.8 | 2.4 | 11.2 |
| Macromoths | 4.8 | 3.5 | 4.3 | 0.5 | 25.2 |
| Non-marine molluscs | 8.8 | 10.0 | 3.6 | 4.0 | 13.6 |
| Shieldbugs and allies | 15.5 | 14.4 | 17.3 | 4.9 | 27.7 |
| Soldierflies and allies | 7.4 | 6.9 | 3.6 | 3.7 | 14.5 |
| Spiders | 4.1 | 3.6 | 2.4 | 1.6 | 10.0 |
| Wasps | 9.6 | 6.7 | 7.8 | 3.4 | 20.1 |
| All groups | 6.9 | 5.3 | 5.4 | 0.5 | 56.9 |

**Table S5.** Summary statistics for changes (%) in the climatic suitability of species' range margins between 1976-1990 and 2001-2015. Positive values indicate an increase in climatic suitability, and negative values indicate a decrease.

| Taxonomic group | Mean | Median | IQR | Min | Max |
|---|---|---|---|---|---|
| Aquatic bugs | 32.4 | 28.4 | 50.6 | -16.2 | 96.7 |
| Bees | 8.6 | 2.8 | 28.3 | -37.9 | 60.9 |
| Butterflies | 1.5 | 2.4 | 31.4 | -44.3 | 59.2 |
| Dragonflies and damselflies | 34.1 | 42.2 | 15.4 | 2.6 | 49.4 |
| Grasshoppers and allies | 13.3 | 10.2 | 22.3 | -31.9 | 72.9 |
| Ground beetles | 30.7 | 34.1 | 19.6 | -3.6 | 55.2 |
| Hoverflies | 15.3 | 7.6 | 27.0 | -17.7 | 104.3 |
| Macromoths | 28.0 | 19.9 | 45.0 | -18.6 | 236.8 |
| Non-marine molluscs | 105.7 | 72.9 | 108.1 | -3.2 | 243.5 |
| Shieldbugs and allies | 8.0 | 8.9 | 19.9 | -16.8 | 51.5 |
| Soldierflies and allies | 12.9 | 11.5 | 17.0 | -15.0 | 41.8 |
| Spiders | 25.4 | 17.6 | 46.7 | -28.7 | 102.2 |
| Wasps | 3.2 | -2.0 | 32.3 | -28.6 | 34.2 |
| All groups | 22.1 | 14.4 | 39.4 | -44.3 | 243.5 |

**Table S6.** Linear mixed-effects models of species' range shifts, with random intercepts on taxonomic group. Fixed effects: habitat specialisation; $log_{10}$-habitat availability at the range margin; exposure to climate change at the range margin (all predictors centred and scaled). Bold text identifies the top models (ΔcAIC < 6), when considering all taxonomic groups (upper) and a subset of groups with high levels of recording (lower).

| SD (Group intercepts) | Species-level effects | | | | | Fit statistics | | |
|---|---|---|---|---|---|---|---|---|
| | Intercept | Habitat specialisation | Habitat availability | Climate exposure | Interaction | $R^2_m$ | $R^2_c$ | cAIC |
| **All taxonomic groups included (n = 291 species, 13 groups)** | | | | | | | | |
| 0.38 | 1.99*** | -0.51** | – | – | – | 0.04 | 0.06 | 1398 |
| **0.76** | **1.73*****| **–** | **1.01***** | **–** | **–** | **0.13** | **0.21** | **1367** |
| 0.06 | 2.04** | – | – | 0.55*** | – | 0.04 | 0.04 | 1398 |
| 0.05 | 2.05** | -0.50** | – | 0.54*** | – | 0.08 | 0.08 | 1389 |
| **0.60** | **1.77***** | – | **0.93***** | **0.39*** | – | **0.14** | **0.19** | **1365** |
| 0.06 | 2.05** | -0.50** | – | 0.52** | 0.07 | 0.08 | 0.08 | 1391 |
| **0.59** | **1.74***** | **–** | **0.93***** | **0.41**** | **0.13** | **0.15** | **0.19** | **1366** |
| **Most consistently recorded taxonomic groups (n = 49 species, 4 groups)** | | | | | | | | |
| 0.77 | 2.09* | -0.62 | – | – | – | 0.04 | 0.11 | 249 |
| 0.00 | 2.06*** | – | 1.40*** | – | – | 0.22 | 0.22 | 238 |
| 0.58 | 2.06* | – | – | 1.18** | – | 0.16 | 0.19 | 244 |
| 0.68 | 2.05* | -0.50 | – | 1.13** | – | 0.19 | 0.24 | 243 |
| 0.00 | 2.06*** | – | 1.14** | 0.83* | – | 0.29 | 0.29 | 235 |
| 0.00 | 1.92*** | -0.44 | – | 1.84*** | -1.15** | 0.30 | 0.30 | 236 |
| **0.00** | **1.61***** | **–** | **1.29***** | **1.57***** | **1.46***** | **0.44** | **0.44** | **224** |

***, P < 0.001; **, P < 0.01; *, P < 0.05

**Table S7.** Species-level results for: rates of range shifts (measured between 1976-1990 to 2001-2015, positive values indicate northward expansion); levels of habitat specialisation (unitless, high values indicate a high level of habitat specialisation), habitat availability in species' 1976-1990 range-margin landscapes, and change in the climatic suitability of species' range margins between 1976-1990 and 2001-2015 (positive values indicate an increase in climatic suitability).

| Taxonomic group | Species | Range shift (km $y^{-1}$) | Habitat specialisation | Habitat availability (%) | Climate exposure (%) |
|---|---|---|---|---|---|
| Aquatic bugs | Corixa panzeri | 1.64 | 1.06 | 6.0 | 8.0 |
| Aquatic bugs | Cymatia coleoptrata | -0.16 | 0.89 | 6.2 | 5.0 |
| Aquatic bugs | Ilyocoris cimicoides | 3.36 | 0.57 | 24.6 | 96.7 |
| Aquatic bugs | Microvelia reticulata | 2.00 | 0.60 | 18.0 | 73.8 |
| Aquatic bugs | Notonecta maculata | 0.72 | 0.84 | 5.9 | 0.2 |
| Aquatic bugs | Notonecta viridis | 5.96 | 0.77 | 10.5 | 28.4 |
| Aquatic bugs | Plea minutissima | 1.44 | 0.46 | 20.2 | 55.5 |
| Aquatic bugs | Ranatra linearis | 5.44 | 1.00 | 8.1 | 39.8 |
| Aquatic bugs | Sigara stagnalis | -2.16 | 1.64 | 5.7 | -16.2 |
| Bees | Andrena bicolor | 5.92 | 0.62 | 14.3 | -7.2 |
| Bees | Andrena denticulata | 6.00 | 1.01 | 6.1 | -2.7 |
| Bees | Andrena dorsata | 1.36 | 0.60 | 16.1 | 21.9 |
| Bees | Andrena flavipes | 2.44 | 0.50 | 17.9 | 26.3 |
| Bees | Andrena helvola | -1.48 | 1.06 | 5.3 | -37.9 |
| Bees | Andrena labialis | 0.04 | 0.98 | 6.0 | 60.9 |
| Bees | Andrena minutula | 3.12 | 0.52 | 13.5 | -5.3 |
| Bees | Andrena nigroaenea | 0.28 | 0.56 | 15.4 | -16.0 |
| Bees | Andrena ovatula | 3.24 | 0.87 | 4.7 | 28.3 |
| Bees | Andrena praecox | 2.76 | 1.03 | 9.2 | 17.1 |
| Bees | Andrena trimmerana | 0.00 | 1.49 | 3.9 | 52.1 |
| Bees | Anthidium manicatum | 7.59 | 1.05 | 6.1 | 9.9 |
| Bees | Anthophora bimaculata | 1.32 | 0.88 | 8.4 | 50.1 |
| Bees | Anthophora furcata | -0.20 | 1.03 | 4.4 | -3.7 |
| Bees | Chelostoma campanularum | 0.32 | 1.25 | 6.7 | 0.8 |
| Bees | Colletes daviesanus | -1.36 | 0.85 | 7.1 | 2.8 |
| Bees | Colletes fodiens | -2.04 | 0.97 | 5.9 | 23.9 |
| Bees | Epeolus cruciger | 1.08 | 1.22 | 6.9 | -8.0 |
| Bees | Epeolus variegatus | 1.12 | 0.80 | 5.9 | 8.4 |
| Bees | Halictus tumulorum | 2.96 | 0.55 | 13.7 | -3.6 |

| Taxonomic group | Species | Range shift (km $y^{-1}$) | Habitat specialisation | Habitat availability (%) | Climate exposure (%) |
|---|---|---|---|---|---|
| Bees | Hylaeus brevicornis | -2.04 | 0.76 | 6.9 | -5.5 |
| Bees | Hylaeus communis | 0.64 | 0.71 | 14.0 | 8.2 |
| Bees | Hylaeus confusus | 0.60 | 1.07 | 5.0 | -6.8 |
| Bees | Hylaeus hyalinatus | 1.96 | 0.88 | 7.6 | 13.6 |
| Bees | Lasioglossum laevigatum | 0.32 | 1.16 | 3.7 | -31.8 |
| Bees | Lasioglossum leucozonium | 2.60 | 0.43 | 12.1 | 2.6 |
| Bees | Lasioglossum minutissimum | 2.08 | 0.75 | 6.6 | 15.5 |
| Bees | Lasioglossum morio | 2.32 | 0.62 | 15.0 | 23.9 |
| Bees | Lasioglossum parvulum | 3.00 | 0.78 | 6.7 | -19.9 |
| Bees | Lasioglossum punctatissimum | 0.20 | 0.91 | 4.6 | -10.2 |
| Bees | Lasioglossum smeathmanellum | 1.32 | 0.74 | 6.1 | 39.3 |
| Bees | Megachile centuncularis | -0.28 | 1.03 | 5.2 | 12.1 |
| Bees | Megachile ligniseca | 4.20 | 1.21 | 6.0 | 32.5 |
| Bees | Melitta tricincta | -0.04 | 1.26 | 10.0 | 2.7 |
| Bees | Nomada flava | 9.95 | 0.59 | 21.3 | -2.7 |
| Bees | Nomada fucata | 1.12 | 0.67 | 7.9 | 21.6 |
| Bees | Osmia leaiana | 0.64 | 0.99 | 5.1 | 19.8 |
| Bees | Panurgus calcaratus | -0.40 | 0.98 | 7.8 | 24.3 |
| Bees | Sphecodes crassus | 4.80 | 0.90 | 5.8 | -3.0 |
| Bees | Sphecodes ephippius | 5.20 | 0.61 | 11.5 | -7.5 |
| Bees | Sphecodes monilicornis | 4.36 | 0.59 | 8.7 | -3.7 |
| Bees | Sphecodes pellucidus | -0.08 | 1.09 | 4.7 | -10.0 |
| Bees | Sphecodes puncticeps | 3.00 | 0.77 | 6.1 | 36.8 |
| Butterflies | Apatura iris | 1.84 | 1.40 | 1.4 | -15.0 |
| Butterflies | Aricia agestis | 3.76 | 0.92 | 7.6 | 8.2 |
| Butterflies | Celastrina argiolus | 5.40 | 0.36 | 15.7 | 13.1 |
| Butterflies | Gonepteryx rhamni | 2.68 | 0.53 | 20.1 | 0.8 |
| Butterflies | Hamearis lucina | -1.60 | 1.61 | 1.8 | -44.3 |
| Butterflies | Hesperia comma | -0.08 | 1.38 | 3.6 | -29.3 |
| Butterflies | Leptidea sinapis | 3.08 | 1.76 | 0.7 | -22.2 |
| Butterflies | Limenitis camilla | 1.84 | 1.44 | 3.6 | 13.0 |
| Butterflies | Melanargia galathea | 3.40 | 0.73 | 20.5 | 4.0 |
| Butterflies | Plebejus argus | -0.04 | 1.61 | 5.5 | 45.8 |
| Butterflies | Polyommatus bellargus | 0.84 | 1.82 | 4.1 | -0.1 |
| Butterflies | Polyommatus coridon | 3.20 | 1.31 | 5.3 | -19.3 |
| Butterflies | Pyrgus malvae | -0.36 | 1.04 | 6.6 | -18.0 |
| Butterflies | Pyronia tithonus | 0.80 | 0.27 | 56.9 | 6.8 |
| Butterflies | Satyrium pruni | 0.48 | 1.63 | 1.3 | 59.2 |
| Butterflies | Thymelicus lineola | 3.64 | 0.74 | 11.6 | 21.7 |
| Dragonflies and damselflies | Aeshna mixta | 6.32 | 0.50 | 23.2 | 39.8 |
| Dragonflies and damselflies | Anax imperator | 10.35 | 0.51 | 22.1 | 43.3 |

| Taxonomic group | Species | Range shift (km $y^{-1}$) | Habitat specialisation | Habitat availability (%) | Climate exposure (%) |
|---|---|---|---|---|---|
| Dragonflies and damselflies | Brachytron pratense | 0.60 | 1.20 | 6.1 | 42.5 |
| Dragonflies and damselflies | Erythromma najas | 1.04 | 0.92 | 8.3 | 42.2 |
| Dragonflies and damselflies | Gomphus vulgatissimus | 0.16 | 0.84 | 6.5 | 2.6 |
| Dragonflies and damselflies | Libellula fulva | 0.04 | 1.63 | 3.8 | 46.5 |
| Dragonflies and damselflies | Orthetrum cancellatum | 8.11 | 0.80 | 13.0 | 49.4 |
| Dragonflies and damselflies | Platycnemis pennipes | 0.64 | 0.78 | 10.3 | 12.3 |
| Dragonflies and damselflies | Sympetrum sanguineum | 6.08 | 0.69 | 11.1 | 27.9 |
| Grasshoppers and allies | Chorthippus albomarginatus | 1.24 | 0.89 | 11.7 | 8.6 |
| Grasshoppers and allies | Conocephalus discolor | 9.11 | 0.76 | 19.9 | 14.3 |
| Grasshoppers and allies | Conocephalus dorsalis | 4.52 | 1.16 | 4.5 | 72.9 |
| Grasshoppers and allies | Labia minor | -1.72 | 1.28 | 1.8 | -19.0 |
| Grasshoppers and allies | Leptophyes punctatissima | 2.96 | 0.69 | 22.8 | 10.0 |
| Grasshoppers and allies | Meconema thalassinum | 1.40 | 0.80 | 12.8 | -17.6 |
| Grasshoppers and allies | Metrioptera brachyptera | 1.88 | 1.91 | 3.5 | 2.8 |
| Grasshoppers and allies | Metrioptera roeselii | 3.48 | 0.73 | 28.4 | 25.0 |
| Grasshoppers and allies | Pholidoptera griseoaptera | -0.88 | 0.56 | 20.4 | 10.4 |
| Grasshoppers and allies | Stenobothrus lineatus | 2.68 | 1.29 | 1.7 | -31.9 |
| Grasshoppers and allies | Tetrix subulata | 3.80 | 0.84 | 7.5 | 18.4 |
| Grasshoppers and allies | Tettigonia viridissima | 1.40 | 0.75 | 3.0 | 66.2 |
| Ground beetles | Bembidion articulatum | 2.64 | 0.89 | 5.8 | 33.8 |
| Ground beetles | Bembidion assimile | 1.04 | 1.07 | 4.4 | 55.2 |
| Ground beetles | Demetrias atricapillus | 0.12 | 0.89 | 8.5 | 34.4 |
| Ground beetles | Leistus spinibarbis | 0.88 | 0.89 | 4.5 | 1.6 |
| Ground beetles | Poecilus cupreus | 2.08 | 0.88 | 6.5 | 45.7 |
| Ground beetles | Pterostichus nigrita | 14.11 | 0.86 | 7.1 | -3.6 |
| Ground beetles | Stenolophus mixtus | 0.44 | 0.99 | 5.4 | 33.4 |
| Ground beetles | Syntomus foveatus | -2.76 | 0.91 | 3.5 | 44.8 |
| Hoverflies | Cheilosia soror | 1.04 | 1.30 | 3.1 | 18.6 |
| Hoverflies | Cheilosia vulpina | -2.04 | 0.76 | 2.4 | -5.6 |
| Hoverflies | Chrysotoxum cautum | -0.28 | 0.58 | 5.4 | -17.7 |
| Hoverflies | Chrysotoxum festivum | 1.32 | 0.75 | 4.1 | 13.8 |
| Hoverflies | Eumerus strigatus | -3.32 | 0.67 | 2.8 | -0.2 |
| Hoverflies | Parhelophilus frutetorum | -1.24 | 0.79 | 2.8 | -7.4 |
| Hoverflies | Parhelophilus versicolor | -3.36 | 1.02 | 3.1 | 1.3 |
| Hoverflies | Tropidia scita | 2.16 | 0.98 | 4.0 | 13.9 |
| Hoverflies | Volucella inanis | 6.68 | 1.03 | 11.2 | 33.2 |
| Hoverflies | Volucella inflata | 2.44 | 1.13 | 2.8 | -4.5 |
| Hoverflies | Volucella zonaria | 5.00 | 1.55 | 6.1 | 104.3 |
| Hoverflies | Xanthogramma pedissequum | 0.36 | 0.62 | 6.3 | 33.5 |
| Macromoths | Acronicta aceris | 2.44 | 0.78 | 6.2 | 15.4 |
| Macromoths | Acronicta tridens | 1.44 | 0.58 | 1.1 | 0.5 |

| Taxonomic group | Species | Range shift (km y$^{-1}$) | Habitat specialisation | Habitat availability (%) | Climate exposure (%) |
|---|---|---|---|---|---|
| Macromoths | Agrotis cinerea | -0.08 | 1.25 | 1.2 | -11.9 |
| Macromoths | Agrotis puta | 6.20 | 0.47 | 11.1 | 9.1 |
| Macromoths | Agrotis ripae | 0.68 | 1.76 | 0.5 | 53.6 |
| Macromoths | Agrotis trux | 1.12 | 0.92 | 1.4 | 76.5 |
| Macromoths | Amphipyra pyramidea | 9.75 | 0.61 | 16.0 | 3.3 |
| Macromoths | Angerona prunaria | 0.16 | 1.10 | 1.9 | 21.1 |
| Macromoths | Apamea sublustris | 2.08 | 1.10 | 3.4 | -8.2 |
| Macromoths | Apoda limacodes | 1.68 | 0.96 | 3.4 | 64.9 |
| Macromoths | Aporophyla australis | 3.40 | 0.98 | 4.4 | 54.6 |
| Macromoths | Archanara dissoluta | 1.32 | 1.17 | 1.9 | 54.2 |
| Macromoths | Arctia villica | 1.80 | 0.74 | 4.1 | 58.3 |
| Macromoths | Arenostola phragmitidis | 1.68 | 1.59 | 3.6 | 37.5 |
| Macromoths | Aspitates ochrearia | 2.64 | 1.02 | 2.3 | 64.4 |
| Macromoths | Bena bicolorana | 5.04 | 0.93 | 3.7 | 4.5 |
| Macromoths | Catarhoe cuculata | -1.32 | 0.73 | 1.6 | 16.0 |
| Macromoths | Catarhoe rubidata | 1.24 | 0.89 | 1.9 | 35.0 |
| Macromoths | Catocala nupta | 4.16 | 0.74 | 7.9 | -1.3 |
| Macromoths | Cepphis advenaria | 1.16 | 1.64 | 1.6 | -0.4 |
| Macromoths | Chilodes maritima | 2.92 | 1.59 | 1.6 | 44.9 |
| Macromoths | Clostera curtula | 0.24 | 0.70 | 5.6 | 4.0 |
| Macromoths | Coenobia rufa | 2.96 | 0.88 | 4.2 | 36.2 |
| Macromoths | Comibaena bajularia | -0.44 | 0.98 | 3.7 | 27.0 |
| Macromoths | Conistra rubiginea | 3.80 | 0.78 | 3.8 | 12.4 |
| Macromoths | Cosmia affinis | -0.80 | 0.78 | 1.4 | 213.1 |
| Macromoths | Cosmia pyralina | -0.20 | 1.02 | 4.7 | 69.0 |
| Macromoths | Cucullia asteris | 1.08 | 1.30 | 1.0 | 236.8 |
| Macromoths | Cyclophora annularia | 0.76 | 1.32 | 2.1 | 52.3 |
| Macromoths | Cyclophora linearia | 3.00 | 1.00 | 2.8 | 19.2 |
| Macromoths | Cyclophora punctaria | 3.24 | 0.80 | 5.4 | 51.9 |
| Macromoths | Deltote pygarga | 5.80 | 0.72 | 9.1 | 27.0 |
| Macromoths | Dypterygia scabriuscula | 1.92 | 0.64 | 3.8 | 21.8 |
| Macromoths | Earias clorana | 4.56 | 1.06 | 2.9 | 53.6 |
| Macromoths | Eilema complana | 2.24 | 0.42 | 14.3 | 14.4 |
| Macromoths | Eilema depressa | 13.07 | 0.78 | 10.6 | 7.7 |
| Macromoths | Eilema griseola | 6.12 | 0.54 | 25.2 | 21.8 |
| Macromoths | Eilema sororcula | 8.23 | 0.77 | 9.1 | 27.1 |
| Macromoths | Elaphria venustula | 2.36 | 1.23 | 1.8 | 4.8 |
| Macromoths | Ennomos autumnaria | 1.04 | 1.10 | 2.8 | 29.0 |
| Macromoths | Ennomos quercinaria | 0.96 | 0.86 | 3.0 | 12.6 |
| Macromoths | Eremobia ochroleuca | 2.08 | 0.58 | 12.3 | 10.6 |
| Macromoths | Eriogaster lanestris | 3.36 | 1.08 | 2.6 | 73.0 |

| Taxonomic group | Species | Range shift (km $y^{-1}$) | Habitat specialisation | Habitat availability (%) | Climate exposure (%) |
|---|---|---|---|---|---|
| Macromoths | Euphyia biangulata | 1.96 | 0.95 | 1.9 | -13.3 |
| Macromoths | Euphyia unangulata | 0.56 | 0.65 | 2.7 | 7.9 |
| Macromoths | Eupithecia haworthiata | 2.44 | 0.78 | 2.7 | 15.6 |
| Macromoths | Eupithecia inturbata | 0.68 | 0.86 | 2.2 | -4.0 |
| Macromoths | Eupithecia simpliciata | 2.76 | 0.73 | 1.7 | 56.8 |
| Macromoths | Eupithecia subumbrata | 3.44 | 0.88 | 1.3 | -6.1 |
| Macromoths | Euplagia quadripunctaria | 6.24 | 0.96 | 9.3 | 53.7 |
| Macromoths | Euproctis chrysorrhoea | 2.88 | 0.58 | 5.8 | 56.6 |
| Macromoths | Gastropacha quercifolia | -4.32 | 1.35 | 2.3 | -8.6 |
| Macromoths | Globia sparganii | 4.60 | 1.58 | 3.0 | 61.5 |
| Macromoths | Hemaris fuciformis | 1.60 | 1.15 | 0.9 | 1.0 |
| Macromoths | Hemistola chrysoprasaria | 1.64 | 0.87 | 5.5 | 20.1 |
| Macromoths | Hemithea aestivaria | 0.24 | 0.55 | 14.0 | 15.6 |
| Macromoths | Horisme tersata | 0.52 | 0.82 | 4.0 | -5.6 |
| Macromoths | Horisme vitalbata | 1.44 | 0.88 | 4.8 | 7.7 |
| Macromoths | Hypena rostralis | 3.96 | 1.45 | 2.4 | 49.7 |
| Macromoths | Hypomecis punctinalis | -1.32 | 0.83 | 4.8 | 11.9 |
| Macromoths | Hypomecis roboraria | -0.64 | 1.25 | 1.8 | 20.2 |
| Macromoths | Idaea fuscovenosa | 0.16 | 0.60 | 4.9 | 55.0 |
| Macromoths | Idaea rusticata | 6.95 | 1.11 | 4.7 | 79.9 |
| Macromoths | Idaea subsericeata | 1.12 | 0.69 | 2.2 | 39.5 |
| Macromoths | Idaea sylvestraria | -2.52 | 1.88 | 1.0 | 23.6 |
| Macromoths | Idaea trigeminata | 0.80 | 1.06 | 6.8 | 44.1 |
| Macromoths | Lacanobia suasa | 2.88 | 1.54 | 1.7 | 29.2 |
| Macromoths | Lacanobia w-latinum | 0.32 | 0.60 | 5.3 | 36.5 |
| Macromoths | Larentia clavaria | -1.80 | 0.70 | 3.5 | 19.5 |
| Macromoths | Laspeyria flexula | 5.16 | 0.71 | 10.9 | -0.9 |
| Macromoths | Lenisa geminipuncta | 7.51 | 0.83 | 3.1 | 47.2 |
| Macromoths | Leucania obsoleta | 2.88 | 1.42 | 1.7 | 61.1 |
| Macromoths | Leucoma salicis | 1.04 | 0.85 | 3.7 | -18.2 |
| Macromoths | Ligdia adustata | 1.08 | 0.78 | 4.7 | 19.7 |
| Macromoths | Lithophane ornitopus | 5.76 | 0.79 | 7.1 | -11.8 |
| Macromoths | Lithophane semibrunnea | 6.04 | 1.10 | 1.4 | 2.8 |
| Macromoths | Lomographa bimaculata | 5.04 | 0.88 | 6.6 | -2.9 |
| Macromoths | Lygephila pastinum | 3.04 | 0.54 | 4.5 | -13.4 |
| Macromoths | Lymantria monacha | 2.92 | 0.75 | 13.7 | 34.9 |
| Macromoths | Macaria alternata | 7.23 | 0.49 | 6.2 | 58.0 |
| Macromoths | Macrochilo cribrumalis | 0.88 | 1.48 | 3.6 | 31.1 |
| Macromoths | Malacosoma neustria | -0.56 | 0.51 | 8.8 | 30.4 |
| Macromoths | Meganola albula | 6.36 | 0.62 | 5.3 | 40.2 |
| Macromoths | Melanthia procellata | 2.88 | 0.96 | 2.9 | 16.3 |

| Taxonomic group | Species | Range shift (km $y^{-1}$) | Habitat specialisation | Habitat availability (%) | Climate exposure (%) |
|---|---|---|---|---|---|
| Macromoths | Miltochrista miniata | 1.16 | 0.63 | 12.5 | 54.7 |
| Macromoths | Mimas tiliae | 3.96 | 0.76 | 7.0 | 4.0 |
| Macromoths | Minoa murinata | -0.60 | 1.81 | 1.2 | -2.0 |
| Macromoths | Mythimna l-album | 6.76 | 0.75 | 6.4 | 97.3 |
| Macromoths | Mythimna pudorina | 4.32 | 1.39 | 3.0 | 19.1 |
| Macromoths | Mythimna straminea | 3.96 | 1.58 | 3.4 | 62.4 |
| Macromoths | Nyctobrya muralis | 2.52 | 0.91 | 4.4 | 54.8 |
| Macromoths | Pachycnemia hippocastanaria | -2.96 | 1.43 | 2.3 | 6.8 |
| Macromoths | Paradarisa consonaria | 7.51 | 1.17 | 1.9 | 8.2 |
| Macromoths | Parascotia fuliginaria | 4.24 | 1.77 | 2.7 | 15.3 |
| Macromoths | Parectropis similaria | 1.20 | 1.21 | 2.7 | 5.1 |
| Macromoths | Philereme transversata | 1.32 | 1.02 | 2.6 | -15.5 |
| Macromoths | Philereme vetulata | 0.40 | 0.80 | 1.9 | -15.9 |
| Macromoths | Photedes fluxa | 0.44 | 1.07 | 1.7 | 13.9 |
| Macromoths | Polymixis flavicincta | 1.08 | 0.90 | 8.1 | 20.7 |
| Macromoths | Polyploca ridens | 2.48 | 0.91 | 9.9 | 21.9 |
| Macromoths | Ptilodon cucullina | 0.52 | 0.65 | 3.0 | -9.2 |
| Macromoths | Schrankia taenialis | 1.16 | 1.04 | 0.8 | 63.9 |
| Macromoths | Scopula emutaria | 0.60 | 1.49 | 1.0 | 49.8 |
| Macromoths | Scopula marginepunctata | 0.56 | 0.65 | 1.7 | 79.5 |
| Macromoths | Simyra albovenosa | 0.56 | 1.77 | 2.6 | 48.1 |
| Macromoths | Sphinx ligustri | 2.68 | 0.61 | 8.7 | 35.0 |
| Macromoths | Sphinx pinastri | 4.80 | 0.77 | 6.3 | 29.4 |
| Macromoths | Stauropus fagi | 0.36 | 0.75 | 7.7 | 7.9 |
| Macromoths | Thumatha senex | 1.40 | 1.30 | 4.1 | 9.9 |
| Macromoths | Tiliacea aurago | 0.80 | 0.58 | 15.6 | 9.6 |
| Macromoths | Timandra comae | 1.64 | 0.54 | 10.4 | 9.4 |
| Macromoths | Watsonalla binaria | 1.80 | 0.57 | 7.5 | 5.5 |
| Macromoths | Watsonalla cultraria | 3.44 | 0.95 | 1.9 | -1.2 |
| Macromoths | Xanthorhoe quadrifasiata | 4.76 | 0.57 | 5.8 | -18.6 |
| Macromoths | Zeuzera pyrina | 1.64 | 0.58 | 5.1 | 4.3 |
| Macromoths | Zygaena trifolii | -2.04 | 1.08 | 2.2 | -6.3 |
| Non-marine molluscs | Acroloxus lacustris | 9.31 | 1.40 | 6.5 | 53.7 |
| Non-marine molluscs | Anisus vortex | 4.56 | 0.89 | 10.0 | 161.8 |
| Non-marine molluscs | Bithynia tentaculata | 7.75 | 1.04 | 13.6 | 72.9 |
| Non-marine molluscs | Planorbis planorbis | 2.12 | 1.06 | 10.0 | 243.5 |
| Non-marine molluscs | Pomatias elegans | 0.88 | 1.07 | 4.0 | -3.2 |
| Shieldbugs and allies | Coreus marginatus | 3.48 | 0.60 | 24.9 | 51.5 |
| Shieldbugs and allies | Coriomeris denticulatus | 4.08 | 1.14 | 6.5 | 7.9 |
| Shieldbugs and allies | Dolycoris baccarum | 1.00 | 0.56 | 21.5 | 9.9 |
| Shieldbugs and allies | Myrmus miriformis | -4.68 | 0.92 | 7.3 | -16.8 |

| Taxonomic group | Species | Range shift (km $y^{-1}$) | Habitat specialisation | Habitat availability (%) | Climate exposure (%) |
|---|---|---|---|---|---|
| Shieldbugs and allies | Palomena prasina | 2.36 | 0.71 | 27.7 | 10.7 |
| Shieldbugs and allies | Troilus luridus | 0.48 | 1.04 | 4.9 | -15.1 |
| Soldierflies and allies | Asilus crabroniformis | 0.12 | 0.76 | 11.7 | -8.4 |
| Soldierflies and allies | Chorisops tibialis | 1.28 | 0.77 | 6.1 | 1.0 |
| Soldierflies and allies | Chrysopilus asiliformis | 1.40 | 0.76 | 8.7 | 23.9 |
| Soldierflies and allies | Dioctria atricapilla | -1.40 | 0.91 | 8.5 | 2.7 |
| Soldierflies and allies | Dioctria baumhaueri | 2.64 | 0.97 | 7.3 | 14.0 |
| Soldierflies and allies | Dioctria linearis | -0.16 | 1.13 | 3.7 | 15.7 |
| Soldierflies and allies | Leptogaster cylindrica | 3.28 | 0.90 | 14.5 | 20.1 |
| Soldierflies and allies | Nemotelus notatus | -2.68 | 1.35 | 4.9 | 41.8 |
| Soldierflies and allies | Odontomyia tigrina | -0.08 | 1.56 | 7.5 | 38.8 |
| Soldierflies and allies | Oxycera nigricornis | -0.88 | 1.42 | 4.6 | -15.0 |
| Soldierflies and allies | Oxycera rara | 1.28 | 0.92 | 5.0 | 12.3 |
| Soldierflies and allies | Oxycera trilineata | 0.04 | 1.40 | 4.4 | 10.3 |
| Soldierflies and allies | Pachygaster atra | 4.00 | 0.82 | 10.4 | 23.5 |
| Soldierflies and allies | Pachygaster leachii | 3.48 | 0.86 | 8.3 | 10.2 |
| Soldierflies and allies | Stratiomys singularior | -0.40 | 0.99 | 6.1 | 10.6 |
| Soldierflies and allies | Tabanus bromius | 1.16 | 1.01 | 6.6 | 4.4 |
| Spiders | Achaearanea lunata | 1.80 | 1.24 | 2.4 | -4.5 |
| Spiders | Agalenatea redii | 3.56 | 0.76 | 4.1 | 37.8 |
| Spiders | Agelena labyrinthica | 2.40 | 0.67 | 8.0 | 30.2 |
| Spiders | Anelosimus vittatus | 7.39 | 0.74 | 7.4 | 17.6 |
| Spiders | Clubiona corticalis | -1.12 | 1.05 | 2.2 | -10.6 |
| Spiders | Clubiona pallidula | 2.44 | 0.89 | 2.7 | -9.3 |
| Spiders | Clubiona subtilis | -1.40 | 0.94 | 2.6 | 15.2 |
| Spiders | Diaea dorsata | 2.16 | 1.72 | 1.6 | -2.9 |
| Spiders | Dictyna latens | 0.80 | 1.00 | 3.1 | 81.8 |
| Spiders | Dictyna uncinata | -0.28 | 0.76 | 9.2 | 32.3 |
| Spiders | Enoplognatha latimana | 4.48 | 0.95 | 10.1 | 60.8 |
| Spiders | Enoplognatha thoracica | 2.20 | 0.83 | 3.6 | 9.0 |
| Spiders | Gibbaranea gibbosa | 0.76 | 1.04 | 2.5 | -9.2 |
| Spiders | Hylyphantes graminicola | 2.52 | 0.91 | 3.7 | 39.2 |
| Spiders | Larinioides sclopetarius | 2.80 | 1.67 | 1.8 | 28.1 |
| Spiders | Lathys humilis | -0.48 | 0.75 | 3.9 | 4.3 |
| Spiders | Mangora acalypha | 0.44 | 1.49 | 2.9 | 16.6 |
| Spiders | Misumena vatia | 0.32 | 1.06 | 4.6 | 62.3 |
| Spiders | Neoscona adianta | 0.56 | 0.84 | 6.1 | 91.0 |
| Spiders | Ozyptila praticola | 3.72 | 1.25 | 3.6 | 0.7 |
| Spiders | Pardosa prativaga | -0.52 | 0.67 | 10.0 | 11.6 |
| Spiders | Philodromus albidus | 2.20 | 0.98 | 5.6 | 49.2 |
| Spiders | Philodromus dispar | 2.76 | 0.71 | 5.3 | -5.6 |

| Taxonomic group | Species | Range shift (km $y^{-1}$) | Habitat specialisation | Habitat availability (%) | Climate exposure (%) |
|---|---|---|---|---|---|
| Spiders | Phrurolithus festivus | 3.24 | 1.13 | 2.9 | 51.5 |
| Spiders | Porrhomma microphthalmum | 1.68 | 1.71 | 3.2 | -28.7 |
| Spiders | Simitidion simile | 1.20 | 1.59 | 2.4 | 44.3 |
| Spiders | Tegenaria silvestris | 5.08 | 1.17 | 1.6 | -9.7 |
| Spiders | Theridion pictum | -0.20 | 1.16 | 1.8 | 44.9 |
| Spiders | Theridion tinctum | 2.44 | 0.99 | 5.0 | -1.3 |
| Spiders | Xysticus kochi | 1.88 | 1.14 | 3.6 | 102.2 |
| Spiders | Zilla diodia | 0.88 | 1.06 | 4.1 | 37.6 |
| Wasps | Ammophila pubescens | 0.96 | 1.40 | 3.4 | -10.5 |
| Wasps | Cerceris arenaria | 2.68 | 0.77 | 13.4 | 18.5 |
| Wasps | Cerceris rybyensis | 1.88 | 0.54 | 20.1 | 34.2 |
| Wasps | Crossocerus cetratus | 1.00 | 1.15 | 5.9 | -12.9 |
| Wasps | Episyron rufipes | 2.12 | 1.05 | 5.8 | 28.6 |
| Wasps | Evagetes crassicornis | -1.04 | 0.96 | 6.3 | -28.6 |
| Wasps | Hedychridium ardens | -0.80 | 0.98 | 7.1 | -10.7 |
| Wasps | Oxybelus uniglumis | 0.60 | 0.62 | 14.4 | 6.5 |

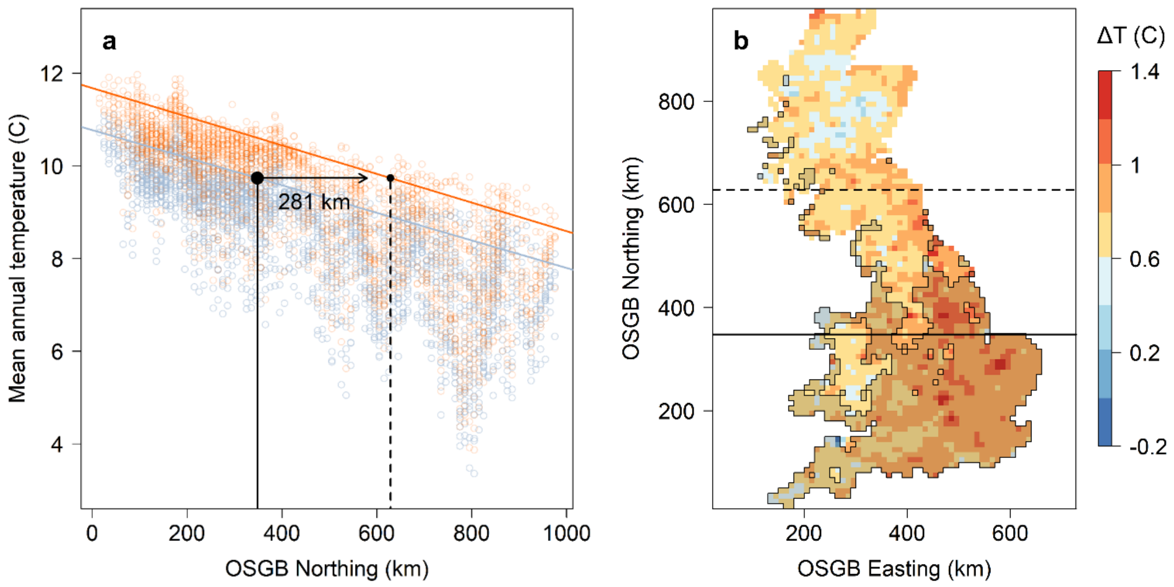


**Figure S1 | Latitudinal shift in mean annual isotherms. a,** Circles show mean annual temperatures of study hectads in the first (1976-1990, blue) and second (2001-2015, orange) recording periods. Regression lines show latitudinal gradients after controlling for elevation in a linear model (latitude coefficient from Temperature ~ Latitude*Elevation). Arrow indicates the latitudinal shift in isotherms, beginning at the median 1976-1990 range margin across the 291 study species (large black dot) and tracking the corresponding temperature northwards into the 2001-2015 recording period (small black dot). **b**, Change in mean annual temperature shown spatially, with isotherm shift marked with lines as in part a. Shaded region shows the warmest 50% of hectads.

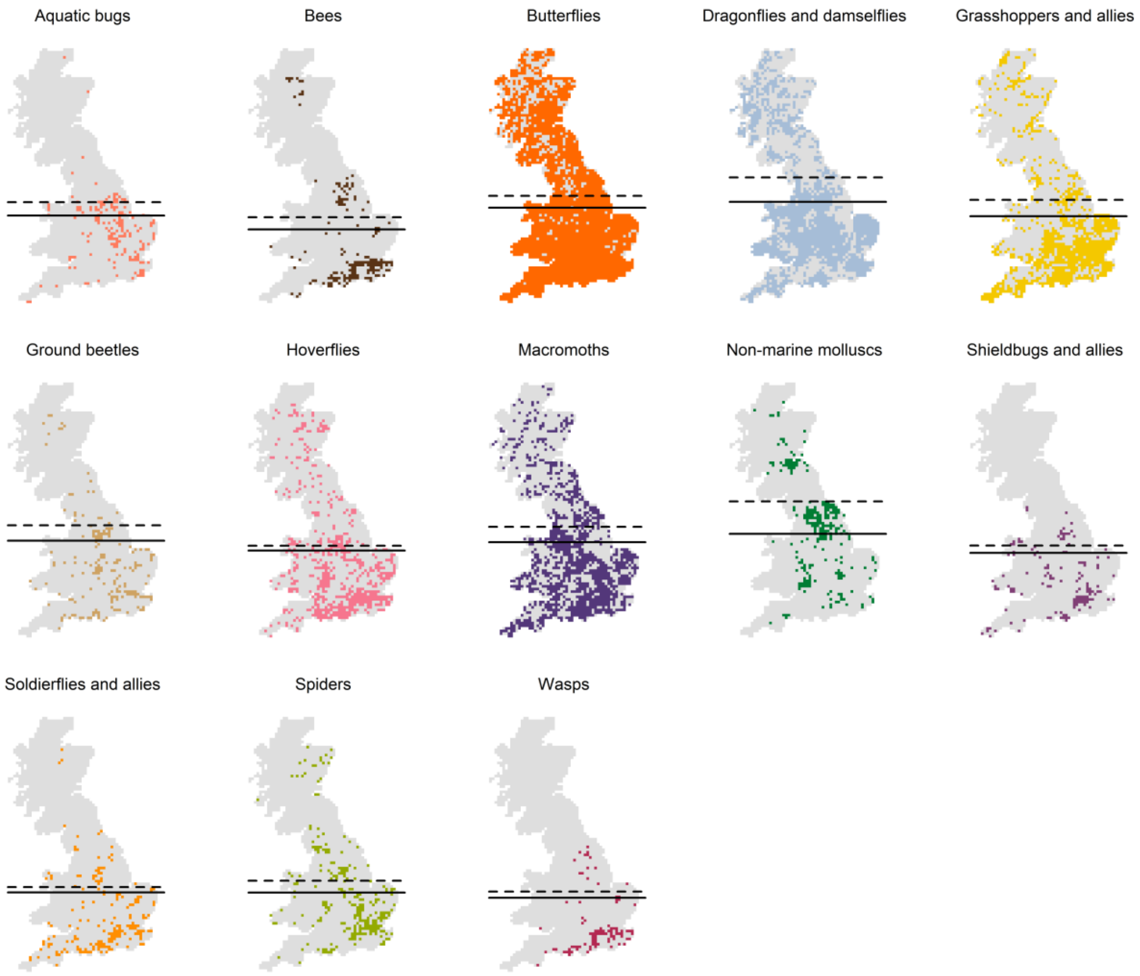


**Figure S2 | Distribution of well-recorded hectads.** Solid and dashed lines show, respectively, mean latitudinal range margins in 1976-1990 and 2001-2015.

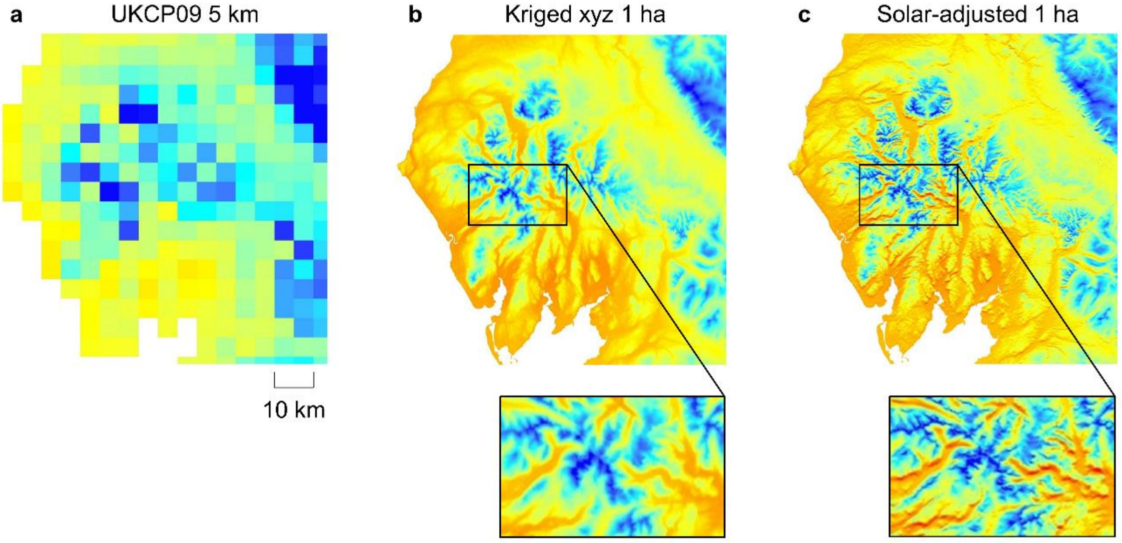


**Figure S3 | Example of climate downscaling.** Spatial variation in degree-days above 5 °C in the UK Lake District, mapped at 5 km × 5 km resolution using data from UKCP09 (a), and at 1-ha resolution (kriging with latitude, longitude and elevation) before (b) and after (c) adjusting for topographic effects on solar radiation.
